# Poly(2‐oxazoline)‐ and Poly(2‐oxazine)‐Based Self‐Assemblies, Polyplexes, and Drug Nanoformulations—An Update

**DOI:** 10.1002/adhm.202001382

**Published:** 2021-01-14

**Authors:** Anna Zahoranová, Robert Luxenhofer

**Affiliations:** ^1^ Institute of Applied Synthetic Chemistry Vienna University of Technology Getreidemarkt 9/163MC Vienna 1060 Austria; ^2^ Functional Polymer Materials Chair for Advanced Materials Synthesis Institute for Functional Materials and Biofabrication Department of Chemistry and Pharmacy Julius‐Maximilians‐Universität Würzburg Röntgenring 11 Würzburg 97070 Germany; ^3^ Soft Matter Chemistry Department of Chemistry Helsinki University Helsinki 00014 Finland

**Keywords:** block copolymers, colloids, cytotoxicity, drug delivery, micelles, microphase separation, thermogelling

## Abstract

For many decades, poly(2‐oxazoline)s and poly(2‐oxazine)s, two closely related families of polymers, have led the life of a rather obscure research topic with only a few research groups world‐wide working with them. This has changed in the last five to ten years, presumably triggered significantly by very promising clinical trials of the first poly(2‐oxazoline)‐based drug conjugate. The huge chemical and structural toolbox poly(2‐oxazoline)s and poly(2‐oxazine)s has been extended very significantly in the last few years, but their potential still remains largely untapped. Here, specifically, the developments in macromolecular self‐assemblies and non‐covalent drug delivery systems such as polyplexes and drug nanoformulations based on poly(2‐oxazoline)s and poly(2‐oxazine)s are reviewed. This highly dynamic field benefits particularly from the extensive synthetic toolbox poly(2‐oxazoline)s and poly(2‐oxazine)s offer and also may have the largest potential for a further development. It is expected that the research dynamics will remain high in the next few years, particularly as more about the safety and therapeutic potential of poly(2‐oxazoline)s and poly(2‐oxazine)s is learned.

## Graphical abstract

Research on poly(2‐oxazoline)s and poly(2‐oxazine)s is gaining momentum in recent years, stimulated by first promising clinical trials and a growing chemical toolbox for both polymer families. This review focuses specifically on recent developments in self‐assemblies and non‐covalent drug delivery systems, with an emphasis on in vivo and in vitro studies.

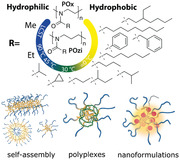

## 1 Introduction

Poly(2‐oxazoline)s (POx) are a long‐known family of synthetic polymers, first synthesized in 1960s.^[^ ^1^, ^2^, ^3^, ^4^ ^]^ POx also have a close relative, poly(2‐oxazine)s (POzi), their higher, and much less investigated, homologue.^[^ ^5^ ^]^ Although providing interesting opportunities for controlled synthesis and plentiful post‐polymerization modifications, they were all but forgotten during the following decades with only few researchers working on this platform. The renaissance of the POx family dates back to the early 2000s, when their potential use as biomaterials in medical applications, namely as an alternative to poly(ethylene glycol), has been (re‐)recognized.^[^ ^6^, ^7^, ^8^ ^]^ Since then, they have been gaining increasing attention in the research community, due to several reasons. They can be prepared by living cationic ring‐opening polymerization (LCROP), providing good control over the resulting (co)polymer structure and properties, narrow dispersity, and access to block copolymers by sequential addition of different monomers. In addition, a 2‐oxazoline monomer containing an additional double bond, 2‐isopropenyl‐2‐oxazoline, can also be polymerized by radical polymerization and anionic polymerization, leaving the 2‐oxazoline ring available for further grafting, bottle‐brush brush synthesis, and other post‐polymerization modifications.^[^ ^9^, ^10^, ^11^ ^]^ Recently, this monomer has also been successfully employed in controlled reversible‐deactivation radical polymerization for the first time, yielding well‐defined polymers with narrow dispersities.^[^ ^12^ ^]^

**Figure 1:**
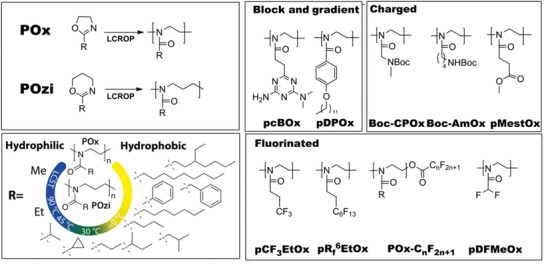
Polymerization of POx and POzi, variability of the solubility profile depending on the side chain of POx/POzi and the overview on the (co)polymers relevant for this review.

The side‐chain substituent of 2‐oxazoline monomer can be varied widely and readily (**Figure** **1**), providing access to a large library of (co)polymers with different solubility, such as hydrophilic, thermoresponsive, hydrophobic, or fluorophilic ones,^[^ ^13^, ^14^, ^15^, ^16^ ^]^ even though for decades, this huge toolkit has not been fully exploited with only a few side chains investigated in the vast majority of papers. Further, various functional groups can be easily introduced into the polymer chain by initiation,^[^ ^17^ ^]^ termination,^[^ ^18^ ^]^ or by using functional monomers.^[^ ^19^ ^]^ In addition to this chemical versatility, POx prepared by LCROP can also exhibit extraordinary low cytotoxicity toward various cell lines, up to the concentration of 10–100 g L^−1^, depending on the (co)polymer composition and the cell type.^[^ ^20^, ^21^, ^22^, ^23^ ^]^ Also, according to several reports, they do not stimulate strong inflammatory response of macrophages in vitro^[^ ^24^ ^]^ and exhibit fast renal clearance in vivo in mice, if the molar mass is sufficiently small.^[^ ^25^ ^]^ Based on these favorable properties, POx have been employed for the preparation of drug conjugates,^[^ ^26^ ^]^ non‐viral gene vectors,^[^ ^27^ ^]^ micelles,^[^ ^28^ ^]^ hydrogels,^[^ ^29^ ^]^ and surfaces with controllable fouling and cell adhesion.^[^ ^30^ ^]^ Although POx have been widely exploited in academic research, the translation into clinical applications has been slow, hindered probably due to the commercial unavailability of high‐quality, narrow dispersity POx for a long time, and the prominent position of poly(ethylene glycol) (PEG)‐based materials on the market. The most noticeable progress in this area has been made by the POx‐conjugated rotigotine for the treatment of Parkinson's disease. The first clinical trial of this conjugate administered subcutaneously in humans started in 2015, with the first promising preliminary results from four subjects being published in 2017.^[^ ^31^ ^]^ More recently, the results from this study showed that the weekly subcutaneous injection of POx‐rotigotine conjugate helps to maintain constant plasma levels of rotigotine in Parkinson's disease patients without reported adverse effects.^[^ ^32^ ^]^

Various biomedical applications of POx have been more exhaustingly described in several review papers.^[^ ^33^, ^34^, ^35^ ^]^ Synthetic strategies to prepare telechelic poly(2‐oxazoline)s were recently nicely reviewed by Delaittre.^[^ ^36^ ^]^ Recent advances in the use of lipopoly(2‐oxazoline)s in liposome technology for drug delivery were recently reviewed by Lapinte and co‐workers and thus, will not be discussed in the present contribution.^[^ ^37^ ^]^ Also, Sedlacek and Hoogenboom summarized the advances in drug delivery systems of POx and POzi, paying special attention to polymer‐drug conjugates. This topic will therefore also not be covered in this review.^[^ ^38^ ^]^ Here, the self‐assembly of thermoresponsive and amphiphilic block copolymers in aqueous solutions is discussed. For other stimuli‐responsive POx‐based materials and their applications, the reader is referred to an extensive recent review by Jana and Uchman.^[^ ^39^ ^]^ For comparison of POx and other types of copolymers for micellar drug delivery, with the emphasis on clinical translation, the reader is referred to a recent comprehensive and excellent review by Kabanov et al.^[^ ^40^ ^]^

Much progress has been made in POx‐ and POzi‐based self‐assemblies and formulations toward understanding of structure‐properties relationship, and translation into in vivo applications. This review aims to cover this progress, with the emphasis of the results published in the last five years. The first part of this review focuses on the self‐assembly of block and gradient POx in water, discussing newly introduced 2‐oxazoline monomers as building blocks, gradient, thermoresponsive, fluorinated, and charged copolymers. The second part of the review covers the POx‐ and POzi‐based polyplexes and drug nanoformulations. The interactions between various drugs and micellar core and corona are discussed. Finally, results from in vitro and in vivo studies of POx and POzi micellar formulations are summarized.

## 2 Self‐Assembled Poly(2‐oxazoline)s

In chemistry, we understand self‐organization or self‐assembly of molecules into (well‐defined) supramolecular structures to be based on non‐covalent or dynamic covalent interactions.^[^ ^41^, ^42^, ^43^ ^]^ Traditionally, polymer self‐assemblies are strongly rooted in and intimately connected with developments in macromolecular engineering and the synthesis of defined block copolymers.^[^ ^44^ ^]^ In the early days of macromolecular engineering, living anionic polymerizations^[^ ^45^, ^46^ ^]^ were the main pillar to access block copolymers for defined self‐assemblies, but in the last decades, well‐controlled reversible deactivation radical polymerizations dominated the field due to much simpler experimental requirements.^[^ ^47^ ^]^ The LCROP of 2‐oxazolines (and 2‐oxazines) has lived for decades in the shadows of these two, much better‐known types of polymerization reactions. This is particularly interesting, as arguably, the LCROP of 2‐oxazolines and 2‐oxazines is much easier to conduct than an anionic living polymerization while it typically gives a better synthetic controlled compared to reversible deactivation radical polymerizations. Admittedly, we may have some bias regarding this assessment. Our bias notwithstanding, it is clear that the LCROP of 2‐oxazolines (and to a lesser extend of 2‐oxazines) has clearly proven more than suitable to synthesize a wide range of block copolymers that allowed the study of self‐assembly. Moreover, by combining monomers with different reactivity in a one‐pot polymerization, gradient copolymers are formed which can exhibit different extends of blockiness and also undergo self‐assembly. For self‐assembly to occur, a sufficient incompatibility of segments of the polymer chain (typically the blocks) must be given. In aqueous solution, this is expressed as a hydrophilic/lipophilic contrast but such solvent selective effects can also be observed in non‐aqueous media. Such self‐assemblies exhibit various morphologies, such as spherical or worm‐like micelles, vesicles and can manifest in changes of macroscopic properties such is turbidity, precipitation, or gelation. The morphology of these self‐assembled structures is, in general, controlled by a balance between two forces, an attractive force between insoluble blocks and a repulsive force between soluble blocks. The balance between these two forces is given by stretching of the polymer chains, interfacial energy between the blocks, interactions among corona‐forming chains, strength of interactions between the blocks, and the volume fraction of each block.^[^ ^48^ ^]^ Since the microphase‐separated polymer chains tend to minimize the interfacial area between the two phases, the resulting morphology depends on the volume fraction of the blocks. When volume fraction of a core‐forming block is relatively small, the block copolymer forms spherical micelles. With increasing of the volume fraction of a core‐forming block, worm‐like micelles or vesicles are preferentially formed.^[^ ^49^ ^]^ In addition to copolymer composition, various other parameters influence the morphology of formed aggregates, for example, copolymer concentration, and the water content and content of (co‐)solvents (if used for the preparation), added ions and homopolymers. Also, the method of self‐assembly influences the aggregation process, which may lead to the formation of various kinetically frozen, non‐equilibrium "intermediate" morphologies. Controlling the above‐mentioned parameters, a wide range of more exotic morphologies apart from spherical micelles, worms and vesicles, such as large compound micelles, bicontinuous rods, inverse rods, large compound vesicles, or multilamellar vesicles. All these considerations are of course relevant for the assembly of block copolymers in general and not specific to POx or POzi. For a more detailed and comprehensive discussion, the reader is referred to an excellent review by Mai and Eisenberg.^[^ ^49^ ^]^ Furthermore, thermoresponsive, charged or fluorinated monomer can be introduced to polymer chains, increasing the complexity of the self‐assembly behavior.

### 2.1 Thermoresponsive Copolymers

The solubility of POx and POzi can be tuned by varying the length of the polymer side chain (Figure 1). While poly(2‐methyl‐2‐oxazoline) (pMeOx) and poly(2‐methyl‐2‐oxazine) (pMeOzi) are fully water‐soluble, poly(2‐butyl‐2‐oxazoline) (pBuOx) and poly(2‐butyl‐2‐oxazine) (pBuOzi), are essentially insoluble in water. POx and POzi with medium length of the side chain (i.e., poly(2‐ethyl‐2‐oxazoline) (pEtOx), poly(2‐ethyl‐2‐oxazine), poly(2‐*n*‐propyl‐2‐oxazine) (pPrOzi), poly(2‐isopropyl‐2‐oxazoline) (piPrOx), poly(2‐*n*‐propyl‐2‐oxazoline) (pPrOx), and poly(2‐cyclopropyl‐2‐oxazoline)) exhibit an LCST (lower critical solution temperature) behavior, that is, they become insoluble and precipitate from the aqueous solution above the critical temperature (cloud point, *T*_cp_).^[^ ^50^ ^]^ The LCST actually refers to the lowest *T*_cp_ in the polymer/solvent phase diagram. For practical reasons, the *T*_cp_ for a particular (co)polymer concentration is more frequently determined research papers but sometimes mistakenly termed the LCST.^[^ ^51^ ^]^ However, the thermoresponsive properties of POx‐based polymers and copolymers go beyond the traditional LCST behavior, including also temperature‐driven self‐assembly, crystallization and gelation, which will be also discussed in this section.

**Figure 2:**
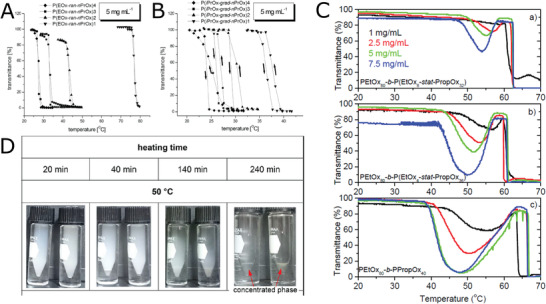
Thermoresponsive POx copolymers. A,B) Transmittance curves during heating and cooling of 5 mg mL^−1^ aqueous solutions of copolymers p(EtOx‐*co*‐PrOx)_stat_, p(iPrOx‐*co*‐PrOx)_grad_. Reproduced with permission.^[^ ^55^ ^]^ Copyright 2016, Elsevier. C) Transmittance curves of pEtOx‐*b*‐p(EtOx‐*co*‐PrOx)_stat_ and pEtOx‐*b*‐pPrOx at various concentrations. Reproduced with permission under the terms of the Creative Commons Attribution 4.0 International License.^[^ ^58^ ^]^ Copyright 2020, the Authors. Published by MDPI. D) Photographs of phase separating aqueous solutions (*w* = 0.0244) of core‐(piPrOx‐*b*‐pEtOx) (left vial in each photograph) and core‐(pEtOx‐*b*‐piPrOx) (right vial in each photograph) heated for prolonged time at 50 °C. Reproduced with permission.^[^ ^64^ ^]^ Copyright 2019, American Chemical Society.

The copolymerization of 2‐oxazolines monomers with either more hydrophilic or hydrophobic co‐monomers leads to thermoresponsive random or gradient coPOx with shifted LCST temperatures, which was already extensively studied by several research groups.^[^ ^13^, ^52^, ^53^, ^54^ ^]^ It should be noted that in principle, not only the composition, but also the distribution of comonomer units along the chain affects the thermoresponsive behavior. That is, the random copolymerization with more hydrophobic or hydrophilic co‐monomer leads to shifting of LCST temperature, while the gradient copolymerization could theoretically lead to the formation of self‐assembled structures with no macroscopically apparent phase separation. This effect of comonomer distribution was studied in the earlier works of Park and Kataoka, when the authors prepared gradient copolymers by the one‐step copolymerization of iPrOx with EtOx^[^ ^13^ ^]^ and iPrOx with PrOx,^[^ ^52^ ^]^ while the one‐pot copolymerization of PrOx and EtOx yielded random copolymers.^[^ ^52^ ^]^ Interestingly, while precise control over LCST temperature was achieved by varying the copolymer composition, no formation of self‐assemblies was observed, even for the gradient copolymers. This work was recently extended by Oleszko‐Torbus et al., where this small library of copolymers was enriched with p(PrOx‐*co*‐MeOx)_grad_ copolymer.^[^ ^55^ ^]^ In addition to heating, the authors also monitored the cooling curves of copolymer aqueous solutions, observing a hysteresis behavior in case of gradient samples, which was not examined previously by Park and Kataoka (**Figure** **2**A,B). Usually, however, the LCST behavior of POx copolymers is fully reversible, unless solutions are heated for prolonged times above the critical temperature.^[^ ^56^, ^57^ ^]^ This hysteresis was attributed by authors to the formation of self‐assembled structures, which was further corroborated by dynamic light scattering (DLS) and atomic force microscopy (AFM) measurements. It should be noted that the formation of aggregates at room temperature (RT) was described also for the copolymer (pPrOx_30_‐*co*‐piPOx_70_)_grad_, although a gradient copolymer with similar composition was previously shown not to form self‐assemblies.^[^ ^52^ ^]^ The thermoresponsive behavior of a more complex block copolymer composed of pEtOx‐*b*‐p(EtOx‐*co*‐PrOx)_stat_ was recently studied by Trinh Che et al.^[^ ^58^ ^]^ (Figure 2C) This work was a follow‐up of an earlier paper from the same group, where the authors described multistep thermoresponsive behavior of such copolymer exhibiting two *T*_cp,_ but only one copolymer concentration was investigated.^[^ ^59^ ^]^ In contrast, several copolymer concentrations were now examined by turbidity measurements and multiangle DLS. The data suggests coexistence of different structures, depending on copolymer concentration and temperature. At low temperatures, at which the samples are clear, unimers coexisted with larger aggregates. In this regime at low concentrations of the copolymer, a third fraction exhibiting very slow relaxation was also present. With increasing temperature, the unimer fraction disappeared in favor of aggregates, reflected in increased turbidity of the solution. At even higher temperatures, presence of smaller micellar structures was observed, presumably leading to increased transmittance of the samples. With further increase of temperature, both blocks collapsed and the samples became turbid again. Interestingly, similar behavior was also observed for a control diblock copolymer pEtOx‐*b*‐pPrOx. In addition to linear diblock copolymers, more complicated copolymer architectures, for example, star‐shaped copolymers, can be achieved by employing multifunctional initiators.^[^ ^14^ ^]^ A small library of star‐shaped gradient and block copolymers p(EtOx‐*co*‐iPOx)_stat/grad_ was recently prepared and their thermoresponsive behavior in water was studied by Kirila, Smirnova et al.^[^ ^60^, ^61^, ^62^ ^]^ The authors prepared 8‐arm star‐shaped copolymer by core‐first method using calix[8]arene (C[8]A) as a core. In contrast to linear analogues, star‐shaped block and gradient copolymers tended to form self‐assemblies already at RT, driven by the aggregation of hydrophobic calixarene cores, as discussed by the authors. When comparing the different comonomer distribution along the polymer chain, not much differences were observed between C[8]A‐p(EtOx‐*co*‐iPrOx)_grad_ and C[8]A‐(pEtOx‐*b*‐piPrOx), while the location of hydrophilic block in the star arm periphery in case of C[8]A‐(piPrOx‐*b*‐pEtOx) led to less sharp phase transition, as judged by light scattering intensity data. It should be noted that the same authors also extensively studied the thermoresponsive behavior of PiPrOx homopolymer based stars with calixarene core and revealed unusually long equilibration times (up to 10 h) of star‐polymers in solution.^[^ ^63^ ^]^ For copolymers‐based stars C[8]A‐(piPrOx‐*b*‐pEtOx), the equilibration time was lower (around 2 h).^[^ ^62^ ^]^ The thermoresponsive behavior of another star‐shaped copolymer composed of four piPrOx‐*b*‐pEtOx arms block was studied by Sato et al.^[^ ^64^ ^]^ The authors compared two different positions of blocks, with piPrOx or pEtOx block attached to the core, respectively. The macroscopic phase transition temperatures and profiles were very similar for the two studied systems. However, core‐(piPrOx‐*b*‐pEtOx) configuration induced micellization of the system, while core‐(pEtOx‐*b*‐piPrOx) formed less‐defined aggregates with heating. The authors also observed liquid‐liquid phase separation after heating the samples at elevated temperature for 4 h (Figure 2D). Such phase separation was also previously reported by the same group in case of linear piPrOx‐*b*‐pEtOx diblock copolymer.^[^ ^65^ ^]^

**Figure 3:**
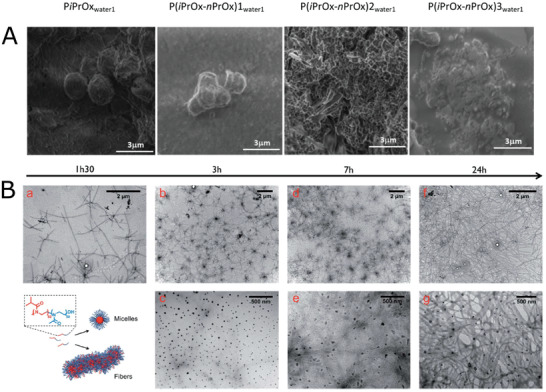
Temperature‐driven crystallization of POx in water. A) Scanning electron microscopy images of homo‐ and copolymers of *i*PrOx incubated in water (1 g L^−1^, 24 h of annealing at 70 °C). Reproduced with permission.^[^ ^71^ ^]^ Copyright 2017, American Chemical Society. B) Transmission electron microscopy (TEM) images of a 10 g L^−1^ of piPrOx‐*b*‐pMeOx solution in water at 65 °C for different times. Reproduced with permission.^[^ ^72^ ^]^ Copyright 2015, The Royal Society of Chemistry.

When the solution of certain POx is kept for prolonged time above its LCST, the polymers can crystallize from the solution. This behavior was for the first time observed and studied in detail for homopolymer PiPrOx in water,^[^ ^66^, ^67^ ^]^ but also some other homopolymers, such as pEtOx in water,^[^ ^68^ ^]^ poly(2‐isobutyl‐2‐oxazoline) (piBuOx) and poly(2‐nonyl‐2‐oxazoline) (pNonOx) in water/ethanol mixtures below their upper critical solution temperature.^[^ ^69^ ^]^ Diehl and Schlaad^[^ ^70^ ^]^ were the first studying the crystallization of random copolymers of iPOx and 2‐(3‐butenyl)‐2‐oxazoline (EnOx). The co‐monomer was selected to allow further functionalization of the crystalline particles. No differences in structure of aggregates were found in comparison to piPOx homopolymers, spherical microparticles made of nanofibers were formed. More recently, Oleszko‐Torbus et al. studied temperature‐induced crystallization of gradient copolymers of iPrOx and PrOx.^[^ ^71^ ^]^ In this study, the presence of PrOx was shown to affect the morphology of formed crystalline particles formed upon prolonged incubation at the temperature above LCST (24 h, 70 °C). In contrast to spherical microparticles composed of fibrillar mesh in case of pure piPOx, the authors observed formation of smooth, smaller spherical crystalline spheres, with a tendency to merge into clusters (**Figure** **3**A). Crystallization of block copoly(2‐oxazoline)s composed of pMeOx‐*b*‐piPOx, was studied by Legros et al.^[^ ^72^ ^]^ While after short (<90 min) annealing above LCST temperature, reversible formation of micelles was observed, for longer annealing times, crystallization occurred. In this case, no individual particles are observed, the copolymer crystallized into a fibrillar network with the presence of spherical nodes (Figure 3B).

**Figure 4:**
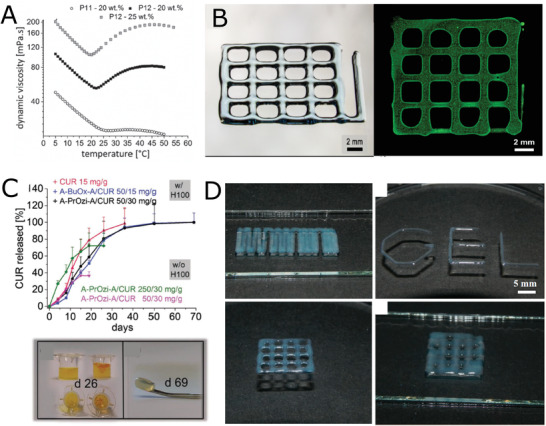
Thermoresponsive block co‐POx and co‐POzi. A) The dependence of viscosity on temperature for thermothickening ABA block copolymers P11 (pMeOx_129_‐*b*‐pPrOx_46_‐*b*‐pMeOx_129_) and P12 (pMeOx_151_‐*b*‐pPrOx_64_‐*b*‐pMeOx_151_) in water. Reproduced with permission.^[^ ^73^ ^]^ Copyright 2017, Wiley‐VCH GmbH. B) Optical microscope image of a printed construct (left) and cell‐loaded constructs (right) from thermogelling diblock copolymer pMeOx‐*b*‐pPrOzi. Reproduced with permission.^[^ ^79^ ^]^ Copyright 2017, American Chemical Society. C) Long‐term curcumin (CUR) release of CUR directly incorporated into pMeOx‐*b*‐pPrOzi hydrogel (20 wt%, red curve) or pre‐incorporated into either pMeOx‐*b*‐pBuOx‐*b*‐pMeOx (polymer/CUR 50/15 mg g^−1^, blue curve) or pMeOx‐*b*‐pPrOzi‐*b*‐pMeOx (polymer/CUR = 50/30 mg g^−1^, black curve) pMeOx‐*b*‐pPrOzi‐*b*‐pMeOx/CUR at either polymer/CUR 250/30 mg g^−1^ (green) or 50/30 mg g^−1^ (pink) without hydrogel for comparison. Appearance of collagen matrix containing hydrogel/pMeOx‐*b*‐pPrOzi‐*b*‐pMeOx/CUR (left) or pMeOx‐*b*‐pPrOzi‐*b*‐pMeOx/CUR 250/30 mg g^−1^ (right) after 26 days incubation in PBS and collagen matrix inicially containing hydrogel/pMeOx‐*b*‐pPrOzi‐*b*‐pMeOx/CUR after release experiment. Reproduced with permission.^[^ ^83^ ^]^ Copyright 2019, Wiley‐VCH GmbH. D) Photographic images of printing experiments using 20 wt% of pMeOx‐*b*‐piBuOx‐*b*‐MeOx. Reproduced with permission under the terms of the Creative Commons Attribution 4.0 International License.^[^ ^86^ ^]^ Copyright 2019, the Authors. Published by MDPI.

Inspired by Pluronic thermogelling triblock copolymers, Zahoranová et al. studied thermoresponsive behavior of ABA and BAB triblock copolymers composed of pMeOx and pPrOx with various ratios of the blocks and various chain lengths.^[^ ^73^ ^]^ Although none of the prepared triblock copolymers exhibited thermogelation (as monitored by development of loss and storage moduli with increasing temperature), an increase of viscosity with temperature (thermothickening) was observed in some of the samples (**Figure** **4**A), particularly ABA copolymers with highest molecular masses (≈30 kg mol^−1^). These copolymers did not exhibit visible clouding, but rather temperature‐induced self‐assembly. Such thermothickening behavior was also described for some other types of LCST (co)polymers, such as polyacrylamide‐based,^[^ ^74^ ^]^ poly(p‐hydroxystyrene)‐graft‐poly(propylene oxide‐*co*‐ethylene oxide)_stat_,^[^ ^75^ ^]^ and polysaccharide‐based copolymers.^[^ ^76^ ^]^

Such materials can find applications also outside the biomedical field, namely in water‐based drilling fluids,^[^ ^77^ ^]^ although much lower copolymer concentrations are usually required for this. In should be noted that Monnery and Hoogenboom^[^ ^78^ ^]^ recently succeeded to prepare thermogelling BAB triblock copolymers pPrOx‐*b*‐pEtOx‐*b*‐pPrOx possessing extraordinarily high molar masses. Interestingly, the gelation was reported to be not reversible. Unfortunately, the authors studied the thermogelling behavior for only one arbitrarily selected concentration of copolymer in water, a more thorough characterization of obtained materials would be interesting.

Furthermore, several other thermoresponsive copolymers with POx and POzi blocks were recently shown to exhibit thermogelation. Lorson et al. recently observed reversible thermogelation of diblock copolymer composed of MeOx and PrOzi.^[^ ^79^ ^]^ Interestingly, the gelation mechanism seems to be different comparing to Pluronic thermogels, as suggested from SANS (small‐angle neutron scattering) measurement. While in case of Pluronic copolymers, the gelation is caused by micelle organization into cubic lattice,^[^ ^80^, ^81^ ^]^ pMeOx‐*b*‐pPrOzi diblock based thermogels exhibit sponge‐like structure formed from merging polymer vesicles.^[^ ^79^, ^82^ ^]^ The copolymer was successfully tested as bioink for biofabrication (Figure 4B) and as an injectable depot for curcumin‐loaded micelles for prolonged drug release in a follow‐up study (Figure 4C).^[^ ^83^ ^]^ For both applications, 3D printing and injectable drug depots, the rheological properties of this hydrogel are well suited. However, for real 3D printing, the yield strength is probably too low and printed strands tend to merge together. Detailed, high shape‐fidelity 3D printing is therefore difficult. Very recently, Hu et al. reported on a significant improvement of the 3D printing and its shape fidelity. This was achieved by adding Laponite XLG, a well‐known viscosity modifier to the thermogelling polymer. Notably, the thermogelation was not strongly affected with only a minor decrease in the gelation temperature and a minor increase in the storage modulus. However, the yield stress was increased considerably and shear‐thinning was enhanced. This led to a considerable improvement of printability of the hydrogel, as evidenced in reduced strand‐fusion and strongly enhance shape fidelity.^[^ ^84^ ^]^ Important to note, it appears that the interaction of clay and pMeOx differs considerably from the interactions of clay and PEG, as very recently studied by Le Coeur et al.^[^ ^85^ ^]^ The authors observed an increase of storage modulus with increasing concentration of pMeOx in pMeOx/Laponite hydrogels above certain critical concentration, while in case of PEG/Laponite, the storage modulus values reached a plateau. This behavior was attributed to stronger adsorption of pMeOx at the clay surface, in comparison to PEG.

Moreover, another triblock copolymer, composed exclusively from POx blocks, exhibited reversible thermogelation, as described by Lübtow et al.^[^ ^86^ ^]^ Specifically, a triblock ABA copolymer composed of MeOx (A) and iBuOx, B was studied. However, the achieved storage modulus was rather low (0.6 kPa in comparison to 4 kPa reported for the pMeOx‐*b*‐pPrOzi diblock by Lorson et al.^[^ ^79^ ^]^), which limits its applicability. In particular, 3D printability suffered from a low yield stress as printed constructs could not hold their shape well (Figure 4D).

During the recent years, several new and interesting thermoresponsive copolymers based on POx or POzi have been described and new block copolymers with thermogelling properties have been introduced, fueled by the renewed interest in such materials in the field of biofabrication (**Table** **1**).^[^ ^87^ ^]^ However, some of these discoveries were more based on trial‐error approach, rather that rational selection of block composition. This is also connected with the fact that the mechanism behind the gelation of some particular types of block copolymers differs from standard model substances such as Pluronic F127 and needs to be clarified in more details.

**Table 1:** Overview on thermoresponsive copolymers discussed in this section; LCST refers to visible clouding of the solution, self‐assembly refers to (temperature driven) formation of smaller aggregates

Copolymer architecture	Composition	Thermoresponsive behavior	Reference
Random copolymers	P(EtOx‐*co*‐PrOx)_stat_	LCST (no self‐assembly)	^[^ ^52^ ^]^
Gradient copolymers	P(EtOx‐*co*‐iPrOx)_grad_	LCST (no self‐assembly)	^[^ ^13^ ^]^
	P(PrOx‐*co*‐iPrOx)_grad_	LCST (no self‐assembly)	^[^ ^52^ ^]^
	P(PrOx‐*co*‐iPrOx)_grad_	LCST, self‐assembly	^[^ ^55^ ^]^
	P(PrOx‐*co*‐iPrOx)_grad_	Crystallization (longer annealing above LCST)	^[^ ^71^ ^]^
	P(MeOx‐*co*‐PrOx)_grad_	LCST, self‐assembly	^[^ ^55^ ^]^
Star‐shaped copolymers	C[8]A‐p(EtOx‐*co*‐iPrOx)_grad_ and C[8]A‐(pEtOx‐*b*‐piPrOx),	LCST, self‐assembly, (long relaxation times)	^[^ ^60^, ^61^ ^]^
	core‐(piPrOx‐*b*‐pEtOx), core‐(pEtOx‐*b*‐piPrOx)	LCST, self‐assembly, (liquid‐liquid phase separation)	^[^ ^64^ ^]^
Block copolymers	pEtOx‐*b*‐pPrOx, pEtOx‐*b*‐p(EtOx‐*co*‐PrOx)_stat_	LCST (two‐step transition), self‐assembly	^[^ ^58^ ^]^
	pMeOx‐*b*‐piPrOx	Self‐assembly, crystallization (longer annealing above LCST)	^[^ ^72^ ^]^
	pMeOx‐*b*‐pPrOx‐*b*‐pMeOx, pPrOx‐*b*‐pMeOx‐*b*‐pPrOx	LCST, self‐assembly, thermothickening	^[^ ^73^ ^]^
	pMeOx‐*b*‐pPrOzi	LCST (*c* < 20 wt%) thermogelation (*c* > 20 wt%)	^[^ ^79^ ^]^
	pMeOx‐*b*‐piBuOx‐*b*‐pMeOx	Thermogelation	^[^ ^86^ ^]^
	pPrOx‐*b*‐pEtOx‐*b*‐pPrOx	Thermogelation	^[^ ^78^ ^]^

### 2.2 Block Copolymers

For decades, research in POx‐based block copolymers concentrated on relatively few building blocks. As hydrophobic monomers, 2‐phenyl‐2‐oxazoline (PhOx) and NonOx were by far the most commonly investigated which by no means reflects the versatility of POx‐based macromolecular engineering (Figure 1). With hindsight, it could be also said that these are probably not the most interesting building blocks. Only very few reports can be found in the literature up to 2010 that deal with other hydrophobic monomers.^[^ ^56^, ^88^, ^89^, ^90^ ^]^ However, in recent years, several new hydrophobic building blocks for POx‐ and POzi‐based block copolymers have been introduced or rediscovered. Traditionally, POx block copolymers are synthesized by a relatively straightforward, one‐pot step‐wise synthesis. In the last decade, some researchers have investigated living polymerization in flow reactors, including microfluidic reactors.^[^ ^91^, ^92^ ^]^ For POx, this was reported by Baeten et al.^[^ ^93^ ^]^ The authors presented the synthesis of triblock copolymers in a microfluidic reactor. Although well‐defined triblock copolymers were successfully synthesized using microfluidic reactor, it should be noted that the reported reaction flow rate was about 1 µL min^−1^, leading to either small batches, or long reaction times comparable to those of conventional CROP.

**Figure 5:**
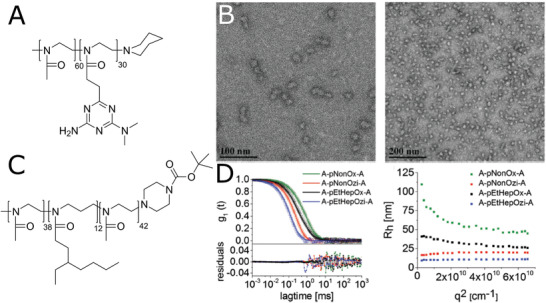
Self‐assembly of block copolymers prepared from novel 2‐oxazoline and 2‐oxazine monomers. A) Chemical structure of diblock copolymer (pMeOx‐*b*‐pcBOx), POx containing newly synthesized hydrophobic monomer. B) TEM image of self‐assembled pMeOx‐*b*‐pcBOx (1 g L^−1^ aqueous solution, stained with uranyl acetate). C) Chemical structure of triblock copolymer pMeOx‐*b*‐pEtHepOzi‐*b*‐pMeOx. D) Left ‐ Autocorrelation function g_1_(t) as well as corresponding fits with residuals of the copolymers at a measurement angle of 90°. The polymer concentration was 10 g L^−1^ (A‐pNonOx‐A: 1 g L^−1^); Right ‐ Apparent hydrodynamic radii of aqueous ABA triblock copolymer solutions as a function of the scattering vector *q*_2_. (B) Reproduced with permission.^[^ ^94^ ^]^ Copyright 2019, Elsevier Ltd. (D) Reproduced with permission.^[^ ^96^ ^]^ Copyright 2018, Wiley‐VCH GmbH.

**Figure 6:**
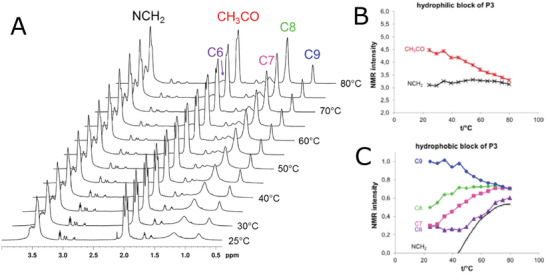
A) ^1^H NMR spectra (in D_2_O) in dependence of the temperature for pMeOx‐*b*‐pNonOx (P3). B) NMR intensities normalized to one proton of the CH_3_CO side group and the NCH_2_ backbone protons of the hydrophilic block of pMeOx‐*b*‐pNonOx. C) NMR intensities normalized to one proton of the CH_2_ and CH_3_ groups of the alkyl side chains and the NCH_2_ backbone protons of the hydrophobic blocks of pMeOx‐*b*‐pNonOx). Reproduced with permission.^[^ ^97^ ^]^ Copyright 2015, American Chemical Society.

Hwang et al.^[^ ^94^ ^]^ prepared an unusual novel diblock copolymer containing a heterocyclic aromatic ring, poly(2‐methyl‐2‐oxazoline)‐*b*‐poly(2‐*N*,*N*‐dimethyl‐1,3,5‐triazine‐2,4‐diamine‐6‐ethyl‐2‐oxazoline) (pMeOx‐*b*‐pcBOx, **Figure** **5**A) by post‐polymerization modification of pMeOx‐*b*‐poly(2‐methoxycarboxyethyl‐2‐oxazoline) (pMestOx). The rather unusual and seemingly complicated structure of the hydrophobic block was designed to interact with encapsulated drug via hydrogen bonding and *π*–*π* stacking. The copolymer self‐assembled in aqueous environment to elongated particles with a diameter of 28 nm (Figure 5B). Encapsulation of various drugs using this copolymer together with in vitro and in vivo evaluation will be further discussed below. Lübtow et al. synthesized triblock ABA copolymers containing newly introduced hydrophobic POzi B blocks poly(2‐(3‐ethylheptyl)‐2‐oxazine) (pEtHepOzi, Figure 5C) and poly(2‐*n*‐nonyl‐2‐oxazine) (pNonOzi), with pMeOx as a hydrophilic block A. In comparison to POx analogues previously introduced by Kempe et al.,^[^ ^95^ ^]^ the POzi copolymers exhibited somewhat lower micellar diameter, below 50 nm (Figure 5D).^[^ ^96^ ^]^ Further, the self‐assembly behavior of block co‐POx was studied in more details, using various techniques. Hiller, Weberskirch et al. studied the effect of temperature on the aggregation of short amphiphilic diblock copolymers (degree of polymerization, DP ≈ 20) composed of hydrophilic MeOx and hydrophobic 2‐pentyl, 2‐heptyl, and NonOx.^[^ ^97^ ^]^ Although the copolymers did not possess a thermoresponsive block, the self‐assembled particles exhibited increase of diameter at the temperature around 40 °C, followed by subsequent gradual decrease. The reason behind this behavior was unfortunately not revealed. The authors further studied the aggregation behavior of the polymers in more detail by ^1^H NMR spectroscopy (**Figure** **6**A–C).

The intensities of protons from selected functional groups in D_2_O (in micellar form) at various temperatures relative to intensities of non‐aggregated form (recalculated from the spectra in non‐selective solvent) were used to quantify the aggregation of the blocks, referred as degree of aggregation. The increased degree of aggregation was observed with increasing length of the side chain of the hydrophobic block, while hydrophilic block did not contribute to the aggregation. This is in line with very early observations by Kabanov on Pluronic block copolymers although in that case, increased hydrophobicity was achieved by increasing of length of the hydrophobic block.^[^ ^98^ ^]^ In addition, the micropolarity of the micellar core was investigated using the pyrene probe in the same work by Weberskirch et al.^[^ ^97^ ^]^ Ratio of intensities of the first and third band of pyrene emission spectrum (*I*_1_/*I*_3_) reflects the polarity of the pyrene microenvironment, with lower values for non‐polar environment. This effect is used for the evaluation of critical micelle concentration (cmc),^[^ ^98^ ^]^ when upon the formation of micelles in solution, pyrene preferentially partitions in the hydrophobic core of the micelle, leading to decrease of *I*_1_/*I*_3_ ratio. The authors observed *I*_1_/*I*_3_ ratios between 1.2 and 1.4, which is in accordance with the values for other types or micelles, such as Pluronics, or many other POx‐based micelles. However, it should be noted that for certain other POx‐based micelles, the measured *I*_1_/*I*_3_ ratios were in fact higher, up to 2.35, exceeding even those observed in aqueous solutions or ionic liquids. This surprising observation was firstly reported by Luxenhofer, Kabanov et al.^[^ ^28^ ^]^ for triblock copolymer of MeOx and BuOx possessing high loading capacity. This finding was subsequently confirmed in other polymers, including one gradient copoly(2‐oxazoline) (vide infra).^[^ ^21^, ^99^, ^100^ ^]^

Self‐assembly of triblock POx‐*b*‐POzi was recently studied with respect to the temperature‐induced gelation. Interestingly, while most of the thermoresponsive POx and POzi represents LCST type (co‐)polymers, which exhibit phase separation with increasing temperature, a different type of thermogel exhibiting inverse thermogelation (gelation upon decreasing the temperature) was recently described by Hahn et al.^[^ ^101^ ^]^ The studied ABA triblock copolymer containing MeOx (A) and poly(2‐phenyl‐2‐oxazine) (PhOzi, B) form a gel with decreasing temperature. The authors revealed that this transition is caused by the change of micellar morphology from worm‐like micelles (below critical temperature) to spherical micelles (above critical temperature). Important to note, this triblock does not possess a thermoresponsive block in its structure and therefore, this transition must rely on some other, currently unknown mechanism. Other, more traditional thermogelling POx and POzi copolymers possessing thermoresponsive block were discussed in the previous section.

### 2.3 Gradient Copolymers

The clear‐cut difference between the different blocks in block copolymers is an important feature for their self‐assembly. However, this clear‐cut difference is not an absolute prerequisite for self‐assembly of copolymers. Another type of copolymers is recently gaining increased attention in this context; gradient copolymers based on POx or POzi. Gradient copolymers are typically formed by one‐pot copolymerization of monomers with different reactivity.^[^ ^102^ ^]^ As fast propagating comonomer, MeOx or EtOx are usually used and yield hydrophilic polymers, with MeOx polymerizing faster than most other 2‐oxazoline with aliphatic side chains. The comonomers which exhibit slower reaction rate are typically those containing a phenyl group directly situated at the 2‐position of the ring, such as PhOx^[^ ^100^ ^]^ or 2‐(4‐dodecyloxyphenyl)‐2‐oxazoline (DPOx).^[^ ^103^ ^]^

**Figure 7:**
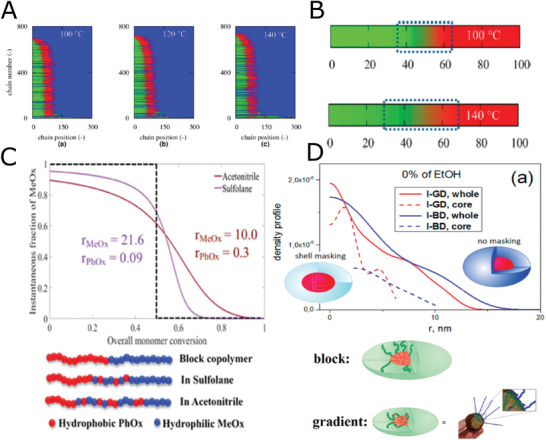
Gradient copolymers based on POx. A) Corresponding final monomer sequences: a) 100, b) 120, and c) 140 °C. Blue: background. B) Theoretical average chains, top; 100 °C and bottom; 140 °C. C) Prediction of the MeOx unit distribution along the copolymer chain based on Skeist's model at f_0_ (MeOx) = 0.5 in the copolymers synthesized in different solvents. Also, the MeOx distribution along the analogues block copolymer chain (black dotted line) is depicted for visual guidance. At the bottom‐right of the figure, a cartoon representation of the obtained monomer distributions is depicted, for visual guidance only. D) Contrast density profiles obtained by deconvolution of the pair‐distance distribution function curves from SANS (up), hypothetical structure of block and gradient nanoparticles and bitterball‐core‐micelles. Red color represents PhOx and green represents MeOx blocks. (bottom). (A, B) Reproduced with permission.^[^ ^105^ ^]^ Copyright 2015, American Chemical Society. (C) Reproduced with permission.^[^ ^108^ ^]^ Copyright 2019, The Royal Society of Chemistry. (D) Reproduced with permission.^[^ ^111^ ^]^ Copyright 2017, American Chemical Society.

Accordingly, the most frequently studied gradient copolymer is p(MeOx‐*co*‐PhOx)_grad_. It should be noted that this copolymer has already been studied earlier with respect to aggregation behavior^[^ ^104^ ^]^ and encapsulation of hydrophobic drug indomethacin.^[^ ^100^ ^]^ In addition to many experimental studies, Hoogenboom et al. recently employed kinetic Monte Carlo model to "visualize" the monomer distribution in p(MeOx‐*co*‐PhOx)_grad_.^[^ ^105^ ^]^ The authors predicted by the model and experimentally confirmed the occurrence of chain transfer reactions, leading to a formation of branched structures (**Figure** **7**A). The authors suggested that the minimal branching can be achieved for lower DP (up to 200), and lower reaction temperatures (100 °C) (Figure 7B). Interestingly, this observation contrasts the earlier reports by the same group suggesting superior polymerization results for higher temperatures (140 °C).^[^ ^106^, ^107^ ^]^ Recently, Hoogenboom et al. also studied the impact of two solvents, acetonitrile and sulfolane, on the monomer distribution along the polymer chain for p(MeOx‐*co*‐PhOx)_grad_.^[^ ^108^ ^]^ While in sulfolane, a quasi‐diblock copolymer was formed, a less steep gradient structure of the copolymer was suggested to form in acetonitrile (Figure 7C). This effect originates in different reactivity ratios of these two monomers in sulfolane in comparison to acetonitrile.^[^ ^109^ ^]^ This nicely highlights the continued importance of understanding of polymerization kinetics. The monomer distribution also affected the size of self‐assembled structures, with largest (hydrodynamic radius, *R*_h_ = 11 nm) micelles observed for gradient copolymers and smallest (*R*_h_ = 5.4 nm) for quasi‐diblock copolymer. The larger size for gradient copolymer‐based self‐assemblies corroborates earlier studies of acrylate/acrylamide based polymers.^[^ ^110^ ^]^

To shed light on the morphology of gradient micelles, Filippov et al. employed X‐ray and neutron scattering techniques to study p(MeOx‐*co*‐PhOx)_grad_ self‐assemblies.^[^ ^111^ ^]^ Notably, the size of gradient micelles was smaller than analogue block copolymers, what is in contradiction with previously discussed paper,^[^ ^108^ ^]^ although in this case, longer polymer chains were synthesized (DP 100 vs DP 50 in ref. ^[^ ^108^ ^]^). Smaller size of gradient p(MeOx_50_‐*co*‐PhOx_50_)_grad_ copolymers in comparison to their diblock analogues in ethanol–water mixtures was also earlier described by Hoogenboom et al.^[^ ^112^ ^]^ More interestingly, in the paper of Filippov et al.,^[^ ^111^ ^]^ the formation of micelles with core‐shell structure was suggested for diblock copolymers, while self‐assemblies with a denser outer layer and a less dense core were observed in case of gradient copolymers (Figure 7D). The authors named such structure "bitterball‐core‐micelles." It would be interesting to study how such structural differences will affect other properties of micelles, such as drug encapsulation ability, stability, and biodistribution.

It is important to point out that the interaction of block copolymers and gradient/random copolymers of an otherwise similar composition with cells differs as previously studied for acrylate/acrylamide based polymers.^[^ ^110^ ^]^ This is most likely connected to the different self‐assembly between the different types of copolymers. However, for POx‐ and POzi‐based systems, this issue has not been studied sufficiently, even though this family of polymers would provide an ideal toolbox to do so.

Very recently, Sedlacek et al. studied the preparation of gradient copolymers from 2‐methyl‐2‐oxazine (MeOzi) and medium chain length aliphatic 2‐oxazolines, namely PrOx and BuOx. Kinetic investigations revealed an interesting phenomenon.

While in homopolymerization, MeOzi shows much slower propagation compared to either PrOx or BuOx, the apparent polymerization rates inverted in copolymerization and MeOzi copolymerized much faster than either PrOx or BuOx. This could be attributed to the crosspropagation rates as the reaction of the more reactive MeOzi with the more reactive 2‐oxazolinium chain ends is strongly favored. This leads to inverted gradient copolymers compared to what might have been assumed from the homopolymerization rates but more importantly, amphiphilic gradient copolymers can still be obtained. Of course, it should be mentioned that also the copolymerization of MeOx with other aliphatic 2‐oxazolines^[^ ^113^ ^]^ or different linear/branched aliphatic 2‐oxazolines can be used to obtain gradient copolymers,^[^ ^52^ ^]^ albeit not as block‐like as is case with PhOx. Interestingly, gradient copolymers of p(MeOzi‐*co*‐PrOx)_*grad*_ showed two different cloud points, one corresponding to pPrOx, the other one to pMeOzi which is slightly more hydrophobic in the presence of PrOx units in the pMeOzi‐rich chain ends.^[^ ^114^ ^]^

### 2.4 Charged Copolymers

Positively charged polymers form polyplexes or polyelectrolyte complexes with negatively charged (macro)molecules. This is heavily employed in particular for complexation of polynucleic acids^[^ ^115^, ^116^ ^]^ (RNA and DNA), intended for use as gene transfer agent, as well as for complexation of negatively charged proteins.^[^ ^117^ ^]^ One of the prototypical polycations for this purpose is poly(ethylene imine) (PEI).^[^ ^118^ ^]^ Essentially, PEI comes in two architectures, linear and branched, which exhibit differences in their toxicity and transfection efficiency. While the branched version is synthesized by ring‐opening polymerization of aziridine, linear PEI is synthesized by complete hydrolysis of POx. However, beyond simply being the precursor of the well‐known PEI, POx can serve as a much more versatile platform to design charged polymers via macromolecular engineering. Irrespective of the desired strategy, free amines cannot directly be incorporated during the polymerization without losing control over the polymerization as such nucleophiles act as terminating reagents of the LCROP. To overcome this issue, two approaches have mainly been employed—either post‐polymerization partial hydrolysis of POx to cationic poly(2‐oxazoline‐*co*‐ethylene imine) (p(Ox‐*co*‐EI)), or copolymerization with monomers bearing protected amino‐groups. In the first approach, typically partial acidic hydrolysis of pEtOx to yield p(EtOx‐*co*‐EI) has been employed. Park et al.^[^ ^27^ ^]^ compared cytotoxicity and transfection efficacy of pEtOx with a high percentage of hydrolysis ranging from 53% to 92%. In that study, the best performing copolymer exhibiting relatively low cytotoxicity against fibroblast cells and highest transfection efficacy was p(EtOx‐*co*‐EI) with 88% of hydrolysis and *M*_w_ of 50 kg mol^−1^. Comparable results were obtained by Fernandes et al.,^[^ ^119^ ^]^ where the authors compared p(EtOx‐*co*‐PEI) with three degree of hydrolysis 30%, 70%, and 96%. There, the authors report acceptable transfection efficacy while maintaining low cytotoxicity for 70% of hydrolysis. In an interesting report, Blankney et al.^[^ ^116^ ^]^ recently showed that for particular types of nucleic acid (plasmid DNA, mRNA, and RepRNA), different *M*_w_ and degree of hydrolysis of pEtOx are preferable. The authors argue that for mRNA, 80% hydrolyzed p(EtOx‐*co*‐EI) is optimal, while for RepRNA and DNA, the best results were obtained with fully hydrolyzed polymer. It should be noted that in addition to pEtOx, a partial hydrolysis of pPrOx^[^ ^120^, ^121^ ^]^ and recently pMeOx^[^ ^122^ ^]^ was also studied. Albeit for a different application, namely for antibacterial effect, Wiesbrock and co‐workers also investigated partially hydrolyzed pNonOx.^[^ ^123^ ^]^

**Figure 8:**
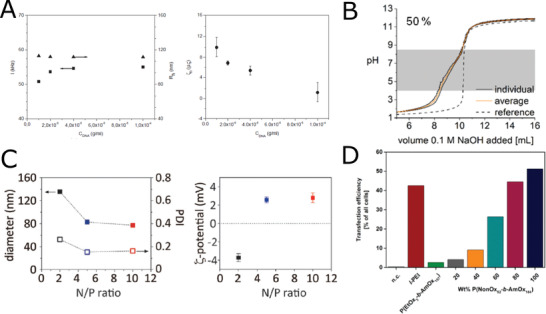
Cationic POx. A) Hydrodynamic radii, and zeta potential of the (p(MeOx‐*co*‐PEI)_stat_‐*co*‐pPhOx)_grad_)/DNA complexes in PBS solutions. Reproduced with permission.^[^ ^125^ ^]^ Copyright 2015, WILEY‐VCH Verlag GmbH & Co. B) Titration curve of p(EtOx‐*co*‐NPrEI) at degrees reduction 51%. Polymers were dissolved in 0.1 m HCl and titrated with 0.1 m NaOH. The titrations were carried out in duplicate or triplicate. The reference titration is in dotted lines, the individual titration curves are shown in black solid lines while the average is red solid lines. Reproduced with permission under the terms of the Creative Commons Attribution 3.0 CC‐BY International License.^[^ ^128^ ^]^ Copyright 2020, the Authors. Published by The Royal Society of Chemistry. C) DLS measurements of particle size and PDI; and zeta potential of polyplexes as a function of N/P ratio of 2 (black), 5 (blue), and 10 (red). Polyplexes were prepared using the CPOx. Reproduced with permission.^[^ ^130^ ^]^ Copyright 2015, WILEY‐VCH Verlag GmbH & Co. D) Transfection efficiency of different polyplexes for adherent HEK‐293 cells in growth media at N*/*P* = 50 after 4 days analyzed via flow cytometry. Values represent the mean. (*n* = 3). Reproduced with permission.^[^ ^131^ ^]^ Copyright 2018, American Chemical Society.

In one of the earlier works on polyplexes forming block copolymers containing hydrolyzed POx, Hsiue et al.^[^ ^124^ ^]^ prepared diblock copolymer by coupling pEtOx with linear p(EtOx‐*co*‐EI) with two different degrees of hydrolysis (content of EI in the diblock 56 and 66 mol%). The diblock copolymers formed polyplexes with plasmid DNA, which were smaller and with almost neutral zeta potential compared to positively charged linear and branched PEI. Moreover, they exhibited lower cytotoxicity and comparable transfection efficacy compared to PEI controls. Unfortunately, the comparison with partially hydrolyzed POx (random copolymers) possessing similar PEI content was not studied in this work. Such comparison would be very interesting to assess the effect of the polymer microarchitecture on the cytotoxicity, polyplex assembly, and transfection efficacy. More recently, Vlassi and Pispas^[^ ^125^ ^]^ also employed the partial hydrolysis approach and prepared partially hydrolyzed gradient copolymers from p(MeOx‐*co*‐PhOx)_grad_. Different rates of hydrolysis of pMeOx and pPhOx blocks, as previously shown by van Kuringen et al.,^[^ ^126^ ^]^ allow preferential hydrolysis of pMeOx block. The self‐assembled structures were thus formed from hydrophobic pPhOx core and hydrophilic and partially charged p(MeOx‐*co*‐EI) corona. The authors also observed the complexation with DNA with the *R*_h_ of complexes 50–100 nm, however, the polyplexes precipitated in a concentration of copolymer higher than 0.15 g L^−1^ (**Figure** **8**A). The cytotoxicity and the transfection efficiency were not studied in this contribution.

Hydrolysis is not the only approach to obtain polycations from POx. POx can also be reduced to yield poly(*N*‐alkyl ethylene imine)s. Interestingly, even though already described decades ago by Saegusa,^[^ ^127^ ^]^ this has been all but forgotten since. More recently, also the partial reduction of POx was described, specifically partial reduction of pEtOx to yield p(EtOx‐*co*‐*N*‐propylethylene imine) (p(EtOx‐*co*‐NPrEI)) was studied. These polymers showed very interesting acid‐base titration curves, that is, almost linear behavior for pH range from 4.5 to 9.5 (Figure 8B). Similar to partially hydrolyzed POx, the cytotoxicity of partially reduced PEtOx was found to be very low for low reduction degrees but quite considerable when the degree of reduction exceeds 30%.^[^ ^128^ ^]^ Therefore, the partially reduced POx appear to be slightly more toxic compared to the corresponding hydrolyzed ones.

In addition to hydrolyzed or reduced POx, cationic charges can be introduced by employing co‐monomer bearing (protected) amino group. POx with pendant amino groups were prepared for the first time by Cesana et al. in 2005.^[^ ^129^ ^]^ As similar approach was used more recently by He et al.,^[^ ^130^ ^]^ where the authors prepared diblock copolymer containing hydrophilic pMeOx and Boc‐protected amine bearing 2‐oxazoline (Boc‐*N*MeMeOx) (Figure 8C). After deprotection, the cationic poly[2‐(*N*‐methyl) aminomethyl‐2‐oxazoline] (p*N*MeMeOx) could be used to form polyplexes. These exhibited low plasma‐protein binding, however, long exposure times (10 h) were required for successful transfection. When comparing these two approaches (partially hydrolyzed gradient copolymer p((MeOx‐*co*‐EI)_stat_‐*co*‐pPhOx)_grad_^[^ ^125^ ^]^ versus block copolymer with deprotected amino group CPOx‐*b*‐MeOx^[^ ^130^ ^]^), both types of copolymers were able to form polyplexes with DNA, although the size of CPOx polyplexes was somehow smaller (*d* = 80 nm vs *d* = 200 nm for p((MeOx‐*co*‐EI)_stat_‐*co*‐pPhOx)_grad_. While p*N*MeMeOx‐*b*‐MeOx copolymer exhibited neutral zeta‐potential even for N/P (nitrogen to phosphorus) ratio up to 10, p((MeOx‐*co*‐EI)_stat_‐*co*‐PhOx)_grad_ displays positive charge, what can be explained by the location of the charge in particle's corona combined with its higher N/P ratio in the range from 20 to 200. Important to note, while in vitro a moderate positive zeta potential might be acceptable, this will lead to multiple problems in vivo, making any application very challenging. As an alternative approach, Schubert et al. developed mixed micelles composed of two different amphiphilic copolymers, a nonionic hydrophobic‐hydrophilic one composed of pNonOx‐*b*‐pEtOx, and a hydrophobic‐polycationic one composed of pNonOx‐*b*‐p[2‐(4‐aminobutyl)‐2‐oxazoline] (pNonOx‐*b*‐pAmOx).^[^ ^131^ ^]^ While the micelles formed from pNonOx‐*b*‐pAmOx alone (without complexed DNA) were spherical, mixing with pNonOx‐*b*‐pEtOx (already at 20 wt% and higher) resulted in rod‐like micelles. Mixing with pNonOx‐*b*‐pEtOx also improved cytocompatibility of the samples, as all mixed copolymers showed reasonably low cytotoxicity up to 0.05 g L^−1^. On the other hand, the highest transfection efficiency was observed for pure pNonOx‐*b*‐pAmOx polyplexes, with the decreasing trend with increasing the pNonOx‐*b*‐pEtOx content. Nevertheless, the transfection efficiency of pure pNonOx‐*b*‐pAmOx block copolymer and pNonOx‐*b*‐pAmOx mixed with 20 wt% of pNonOx‐*b*‐pEtOx were comparable or even higher than transfection efficiencies of control linear PEI (Figure 8D), showing that this concept may have some promise.

Apart from positive charges, negative charges can also be introduced into POx structure, for example, by copolymerization with 2‐methylcarboxyethyl‐2‐oxazoline (MestOx), followed by subsequent hydrolysis yielding carboxyl‐functionalized POx. It should be noted that the synthesis and polymerization of this monomer was introduced already in 1968 by Levy and Litt^[^ ^132^ ^]^ and since then, it was further employed by several research groups.^[^ ^133^, ^134^, ^135^, ^136^ ^]^ The self‐assembly of double‐responsive diblock and triblock copolymers composed of piPOx and poly(2‐carboxyethyl‐2‐oxazoline) (CEtOx) was recently studied by Zschoche et al.^[^ ^137^ ^]^ Depending on the copolymer composition, the authors described formation of self‐assembled nanostructures triggered by the change of pH or temperature. In general, at low temperatures, the copolymers were fully soluble in water, while at temperatures above 60–62 °C the formation of self‐assembled aggregates of various size was observed. Diblock copolymers containing longer pCEtOx block (DP ≥ 30) formed smaller aggregates. The authors identified the smaller aggregates (*R*_h_ ≈ 30 nm) as micelles, while the bigger aggregates were assumed to be vesicles (*R*_h_ > 100 nm). Also triblock copolymers with short flanking pCEtOx block were assumed to form vesicles upon heating. Surprisingly, the effect of the pH was not systematically studied. Only one selected pH value, different for each diblock or triblock system, was discussed in the paper.

To sum up, the self‐assembly of poly(2‐oxazoline)s composed of ionizable groups have been recently employed in the preparation of polyplexes with DNA, where the hydrophilic block adds stealth behavior and increased biocompatibility of formed polyplexes, however, with negative effect on transfection efficacy. On the other hand, introduction of carboxylic group on POx side chain led to the formation of pH responsive nanostructures. Generally, such negatively charged polymers are of interest for the complexation of, for example, positively charged proteins.

### 2.5 Fluorinated Copolymers

Block copolymers containing fluorinated blocks are gaining attention due to potential application as contrast agents for ^19^F magnetic resonance imaging (MRI). The combination with hydrophilic block ensures necessary water‐solubility of the fluorinated contrast agent, for which POx appear as suitable choice. Further combination with lipophilic POx monomer leads to a formation of more complicated multicompartment self‐assembled structures, since lipophilic and fluorophilic blocks of sufficient size are immiscible.^[^ ^138^, ^139^ ^]^

Fluorine can be introduced into POx chain by using fluorinated 2‐oxazoline monomer. However, perfluorinated 2‐oxazoline possesses very low reactivity,^[^ ^140^ ^]^ due to the electron withdrawal effect of perfluoroalkyl moiety, which leads to exceedingly long polymerization times. To overcome this drawback, an alkyl spacer can be placed between the oxazoline ring and fluorine substituted moieties. Accordingly, the polymerization of fluorinated 2‐oxazoline monomer with ethyl spacer, namely 2‐(1H,1H′,2H,2H′‐perfluorohexyl)‐2‐oxazoline, was described by Papadakis, Jordan et al., however, without a detailed study of polymerization kinetics.^[^ ^138^ ^]^ More recently, Filippov et al. studied the polymerization kinetics of a small series of monomers containing CF_3_ group, directly connected to 2‐oxazoline ring, or separated by methyl or ethyl spacer, respectively.^[^ ^141^ ^]^ Presence of the spacer indeed increased polymerization rate. 2‐(3,3,3‐trifluoropropyl)‐2‐oxazoline (CF_3_EtOx), a monomer with an ethyl spacer, exhibited first‐order polymerization kinetics. Further, the authors used the fast‐reacting fluorinated monomer for the preparation of diblock copolymers and triblock terpolymers with MeOx and 2‐octyl‐2‐oxazoline (OctOx). The diblock copolymers self‐assemble in water into smaller particles with hydrodynamic diameters around 20 nm. On the other hand, the size distribution of triblock terpolymers in water measured by DLS was bimodal, with peaks around 30–50 nm and 150–300 nm. Cryo‐TEM revealed presence of particles with spherical shape, with no formation of vesicles observed in this case.

In the development of polymeric MRI contrast agents, low fluorine content and restricted mobility of fluorinated segment, leading to too short spin‐spin relaxation times, remain important challenges in translation into clinics.^[^ ^142^ ^]^ To increase the fluorine content in the fluorinated POx, the same group prepared another fluorinated 2‐oxazoline monomer, 2‐(1H,1H,2H,2H‐perfluorooctyl)‐2‐oxazoline (R_f_^6^EtOx).^[^ ^15^ ^]^ Diblock copolymers and triblock terpolymers composed of R_f_^6^EtOx, hydrophilic MeOx and lipophilic OctOx were prepared and their self‐assembly was studied. In case of diblock copolymers, a bimodal size distribution was observed, with both smaller (*R*_h_ ≈ 10–20 nm) and larger particles present. Cryo‐TEM measurements revealed that the larger aggregates were vesicles. The number of vesicles depended on preparation method, with higher number being formed by direct dissolution, in contrast to solvent exchange method. In case of triblock copolymers, containing an additional lipophilic part, only small spherical particles (*R*_h_ ≈ 15 nm), presumably micelles, were formed. The authors also examined the self‐assembly behavior in the range of organic solvents, and found the formation of particles (*R*_h_ ≈ 50–100 nm) in DMSO. Interestingly, the SANS measurements in DMSO‐d_6_ yielded somehow contradicting results, proving the presence of particles only in case of triblock copolymers.

**Figure 9:**
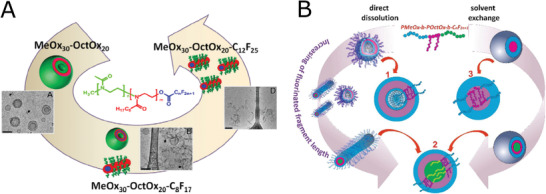
Fluorinated POx. A) Phase sequence of p(MeOx‐*b*‐OctOx) copolymer with increasing the length of terminal perfluoroalkyl chain. Reproduced with permission.^[^ ^144^ ^]^ Copyright 2016, Elsevier. B) A proposed scheme of morphological transition of the fluorinated pMeOx_30_‐*b*‐pOctOx_20_‐C*_n_* F_2_ *_n_*_+1_ nanoparticles (prepared by direct dissolution and by solvent exchange method) with increasing length of fluorinated fragment summarizing the SAXS and SANS results, where: 1—scheme of the inner structure of bilayered vesicle formed by pMeOx_30_‐*b*‐pOctOx_20_ and pMeOx_30_‐*b*‐pOctOx_20_‐C_10_F_21_; 2—scheme of the inner structure of wormlike micelle and core–shell–shell sphere formed by pMeOx_30_‐*b*‐pOctOx_20_‐C*_n_* F_2_ *_n_*_+1_; 3—scheme of the sphere with core–shell inner structure. Reproduced with permission.^[^ ^145^ ^]^ Copyright 2018, Elsevier Ltd.

Another strategy to introduce fluorinated moiety consists of end‐chain attachment of fluorinated segment onto POx by termination or initiation step. In an earlier work, Weberkirch et al.^[^ ^143^ ^]^ used fluorinated initiators to introduce fluorinated moiety into polymer chain. Recently, Filippov et al. prepared quasi‐triblock copolymers from pMeOx‐*b*‐pOctOx by termination with various perfluorinated carboxylic acids.^[^ ^144^ ^]^ In aqueous solution, the authors observed formation of various self‐assembled structures, depending on the length of perfluoroalkyl chain and preparation method (**Figure** **9**A). Diblock copolymers without fluorinated termini formed multi‐layered vesicles (*R*_h_ = 30–60 nm), while in case of copolymers containing fluorinated terminus, rod‐like micelles are prevailing. In a follow‐up paper,^[^ ^145^ ^]^ the authors studied the self‐assembly behavior and internal structure of formed self‐assemblies by cryoTEM, SAXS (small‐angle X‐ray scattering), and SANS. The previously reported effect of preparation method was confirmed also in this paper (Figure 9B). In case of solvent exchange method, only spherical micelles were obtained. On the other hand, direct dissolution method yielded vesicles and rod‐like micelles. Interestingly, also for diblock copolymer without fluorinated segment, the scattering data were fitted with a core–shell–shell model. However, it should be pointed out that the similar core–shell–shell morphologies were also suggested more recently for simple ABA type triblock copolymers without any fluorophilic blocks, albeit in the presence of a hydrophobic drug.^[^ ^146^ ^]^

Yet another approach to introduce fluorinated moiety into POx chain was very recently presented by Sedlacek et al.^[^ ^147^ ^]^The fluorinated copolymers were prepared by partial hydrolysis and subsequent reacetylation using difluoroacetic anhydride, yielding (p(MeOx‐*co*‐2‐difluoromethyl‐2‐oxazoline)_stat_ (p(MeOx‐*co*‐DFMeOx)_stat_) The obtained copolymers were soluble in water at the concentration 50 g L^−1^ up to 34 mol% of fluorinated monomer. The copolymers did not exhibit self‐assembly behavior, as studied by DLS. The copolymers were successfully tested as ^19^F MRI contrast agents in vitro and in vivo in a rat, proving a promising potential for further future applications.

In summary, in recent years, Filippov and co‐workers presented a small series dealing with various strategies of preparation of block coPOx containing fluorinated segments and studied self‐assembly behavior of such copolymers. The authors observed that the position of fluorinated segment influence the morphology of the formed particles. If the fluorinated moieties were in the side chain of POx, spherical particles or vesicles were formed. In contrast, POx with fluorinated termini self‐assembled also into worm‐like micelles. Further, the authors consistently described the impact of preparation method on the self‐assembly of copolymers, where the solvent‐exchange method yielded smaller spherical micelles, while direct dissolution resulted in increased formation of vesicles or worm‐like structures. Possible explanation is that solvent‐exchange method is thermodynamically controlled process, leading to formation of aggregates closer to equilibrium, while the thin film hydration may allow only limited copolymer reorganization, leading to formation of more complex kinetically frozen copolymer assemblies.^[^ ^148^ ^]^ However, this of course strongly depends on the molecular mobility in the aggregates.

Overall, many different strategies have been employed in recent years to prepared self‐assembled POx (and to some extent, POzi), driven by their potential applicability as drug carriers, MRI contrast agents, gene transfer agents, or stimuli‐responsive materials as summarized in **Table** **2**. Block copolymers have been the most widely studied class of copolymers, with several new monomers recently introduced. We are confident this trend of introducing new building blocks will continue in following years, with an emphasis on POzi‐based blocks, which have seen little attention in the past but clearly offer much to discover.

**Table 2:** Comparison of a selection of various cPOx and POzi discussed in this contribution

Type of copolymer	Composition	Self‐assembly morphologies	Suggested application	Reference
Block	pMeOx‐*b*‐poly(2‐*N*,*N*‐dimethyl‐1,3,5‐triazine‐2,4‐diamine‐6‐ethyl‐2‐oxazoline)	Worm‐like micelles	Drug delivery	^[^ ^94^ ^]^
	pMeOx‐*b*‐poly(2‐(3‐ethylheptyl)‐2‐oxazine)‐*b*‐pMeOx, pMeOx‐poly(2‐*n*‐nonyl‐2‐oxazine)‐pMeOx	n.d.	Drug delivery	^[^ ^96^ ^]^
	pMeOx‐*b*‐poly(2‐pentyl‐2‐oxazoline),pMeOx‐*b*‐poly(2‐heptyl‐2‐oxazoline), pMeOx‐*b*‐poly(2‐nonyl‐2‐oxazoline)	n.d.	Micellar catalysis	^[^ ^97^ ^]^
	pMeOx‐*b*‐poly(2‐phenyl‐2‐oxazine)‐*b*‐p(MeOx)	Worm‐like micelles (below *T*_cp_)—spherical micelles (above *T*_cp_)	Bioink	^[^ ^102^ ^]^
Gradient	p(MeOx‐*co*‐PhOx)_grad_	"Bitterball‐core" micelles (higher DP)	Drug delivery	^[^ ^105^, ^108^, ^111^ ^]^
Charged	pEtOx‐*b*‐p(EtOx‐*co*‐EI)_stat_	n.d.	Transfection of DNA	^[^ ^124^ ^]^
	p(MeOx‐*co*‐EI)_stat_‐*co*‐pPhOx)_grad_	n.d.	Transfection of DNA	^[^ ^125^ ^]^
	pMeOx‐*b*‐poly[2‐(*N*‐methyl) aminomethyl‐2‐oxazoline]	Spherical morphology	Transfection of DNA	^[^ ^130^ ^]^
	pNonOx‐*b*‐pEtOx, pNonOx‐*b*‐p[2‐(4‐aminobutyl)‐2‐oxazoline] (mixture)	Spherical or worm‐like micelles	Transfection of DNA	^[^ ^131^ ^]^
	p*i*POx‐*b*‐poly(2‐carboxyethyl‐2‐oxazoline) (AB diblock, ABA, BAB triblocks)	Micelles, polymersomes (vesicles)	Stimuli‐responsive materials (pH, temperature)	^[^ ^137^ ^]^
Fluorinated	pMeOx‐*b*‐2‐(3,3,3‐trifluoropropyl)‐2‐oxazoline, pMeOx‐*b*‐pOctOx‐2‐(3,3,3‐trifluoropropyl)‐2‐oxazoline	Micelles, larger spherical aggregates	MRI contrast agent, drug delivery	^[^ ^141^ ^]^
	pMeOx‐*b*‐poly(2‐(1H,1H,2H,2H‐perfluorooctyl)‐2‐oxazoline), pMeOx‐*b*‐pOctOx‐*b*‐p((1H,1H,2H,2H‐perfluorooctyl)‐2‐oxazoline)	Micelles, polymersomes (vesicles)	MRI contrast agent	^[^ ^15^ ^]^
	pMeOx‐*b*‐pOctOx, pMeOx‐*b*‐pOctOx‐C*_x_* F_2_ *_x_*_+1_	Worm‐like micelles, multilayered vesicles, core–shell–shell micelles	MRI contrast agent	^[^ ^144^, ^145^ ^]^
	(pMeOx‐*co‐*2‐difluoromethyl‐2‐oxazoline)_stat_	No self‐assembly	MRI contrast agents	^[^ ^147^ ^]^

In comparison to block copolymers, gradient copolymers are prepared by somehow easier one‐step synthesis and, as recently shown, exhibit different self‐assembled morphology (smaller bitterball‐core micelles). However, if this different micellar morphology leads to different or even improved performance of such drug carriers in vitro, and more importantly, in vivo, remains to be shown. At this point, they do not seem to be able to compete with block copolymers in this context. Positively charged block copolymers were shown to provide some benefits over traditionally used random partially hydrolysed POx, especially the self‐assembly of such block copolymers increased the biocompatibility of the resulting gene transfer agents. Several fluorinated POx with various architecture and position of fluorinated group have been recently introduced, presenting interesting platform to study the correlation between the chemical structure of copolymers and self‐assembly morphologies. In addition, the importance of preparation method has been emphasized, it would be beneficial if this parameter will be more extensively studied also in context of other types of copolymers.

## 3 Poly(2‐oxazoline)‐Based Nanoformulations

### 3.1 Drug Encapsulation

Self‐assembled block copolymers are well known to be able to solubilize hydrophobic drugs. It is typically assumed that the drug mainly partitions into the core of formed particles. Such solubilization is crucial for many drugs or potential drugs, as a large proportion of natural compounds as well as compounds from high‐throughput screening with biological activity are poorly water soluble. For example, many anti‐cancer drugs exhibit a prohibitively low water solubility, and in order to reach therapeutically relevant concentrations in blood, formulation is required. Commonly employed excipients are Tween or Cremophor EL (now rebranded to Kolliphor EL) but safety concerns exist, in particular activation of the complement system is a potential problem for amphiphilic systems. Block and gradient copoly(2‐oxazoline)s can represent safer alternatives to increase the solubility of such drugs. When designing such polymeric excipients, many researchers try to increase the hydrophilic/lipophilic contrast, in order to increase micellar stability. Also, it is hoped that this would increase drug loading. However, for POx‐based block copolymers it was shown repeatedly that polymers with a minimal hydrophilic/lipophilic contrast, in particular those featuring the barely hydrophobic BuOx, are able to encapsulate extraordinarily high amounts, for example of the anticancer drug Paclitaxel (PTX).^[^ ^28^ ^]^ Since then, POx‐based block copolymer amphiphiles were studied for formulation of large variety of drugs and drug combinations^[^ ^149^ ^]^ and extensive structure‐properties relationship were established.

#### 3.1.1 Interactions of Drugs with Micellar Core

**Figure 10:**
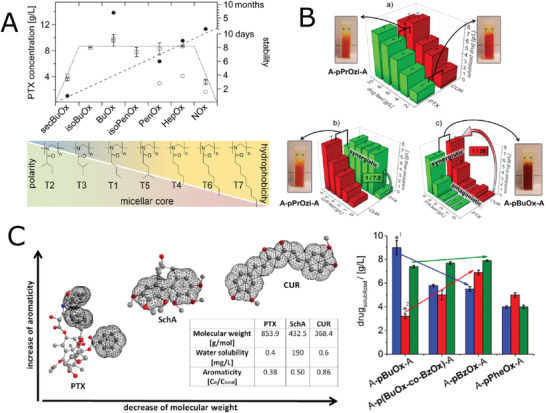
POx and POzi drug formulations. A) Effect of chemical composition of the hydrophobic block on the maximum PTX solubility observed with poly(2‐oxazoline)s (POx) micelle formulations prepared at 0.1 mL scale (squares) and stability over time (filled circles) of POx micelle formulations prepared at 1.5 mL scale at different PTX concentration as shown in the figure (empty circles) in phosphate buffered saline at RT. Reproduced with permission.^[^ ^150^ ^]^ Copyright 2015, John Wiley & Sons, Ltd. B) Coformulated aqueous drug concentrations in dependence of the drug feed concentration (PTX, green bars; CUR, red bars). At a certain drug feed concentration, both drugs were solubilized within a single formulation (≡ coformulation). Data is given as means ± SD (*n* = 3). a) PTX and CUR were added at same feed concentrations and solubilized with A‐pPrOzi‐A (*ρ* = 10 g L^−1^); b) addition of increasing CUR feed concentrations (*ρ* (PTX) = 8 g L^−1^; *ρ* (A‐pPrOzi‐A) = 10 g L^−1^); c) addition of increasing PTX feed concentrations (*ρ* (CUR) = 8 g L^−1^; *ρ* (A‐pBuOx‐A) = 10 g L^−1^). Reproduced with permission.^[^ ^153^ ^]^ Copyright 2017, American Chemical Society. C) Comparison of the hydrophobic drugs PTX, SchA, and CUR with regard to aromaticity, solubility, and molecular weight (left), maximum solubilized aqueous drug concentrations in formulation with the drug carriers (A‐pBuOx‐A, A‐p(BuOx‐*co*‐BzOx)‐A, A‐pBzOx‐A, A‐pPheOx‐A). Maximum solubilization (LE minimum 79%) of the drugs PTX (blue), CUR (red), and SchA (green) using the four polymers. *1 were taken from ref. ^[^ ^18^ ^]^; *2 were taken from ref. ^[^ ^25^ ^]^. In all cases, the polymer concentration was fixed at 10 g L^−1^. Data are given as means ± SD (*n* = 3) (right). Reproduced with permission.^[^ ^158^ ^]^ Copyright 2018, American Chemical Society.

The first study utilizing the ABA triblock copolymers pMeOx‐*b*‐pBuOx‐*b*‐pMeOx included a control polymer with a stronger hydrophilic/lipophilic contrast (pMeOx‐*b*‐pNonOx), which showed inferior drug solubilization but a more detailed structure–properties relationship was not performed.^[^ ^28^ ^]^ Seo and Schulz et al. studied the influence of hydrophobicity of the central 2‐oxazoline core‐forming block on encapsulation efficiency of different taxanes. The authors prepared a series of linear and branched aliphatic POx with various chain lengths (C4–C9) and one aromatic 2‐benzyl‐2‐oxazoline.^[^ ^150^ ^]^ Interestingly, the highest loading capacity (around 44 wt%, maximum solubilization of PTX 9.6 ± 0.9 g L^−1^) was still observed for mildly hydrophobic BuOx, but more generally speaking, the moderately hydrophobic blocks outperformed those with higher or lower hydrophobicity in terms of drug loading (**Figure** **10**A). Interestingly though, in terms of formulation stability at maximal drug loading (here investigated as the colloidal stability of the drug loaded micelles in aqueous media) was higher for highly hydrophobic pMeOx‐*b*‐pNonOx‐*b*‐pMeOx compared to most other formulations. Only pMeOx‐*b*‐pBuOx‐*b*‐pMeOx was the exception, which showed highest drug loading and formulations stability. Quite strikingly, the formulations were colloidally stable for several months despite the extraordinary high drug loading. This study also suggests that branched side chains were less favored, especially if the branches were very close to the amide moiety, that is, the polymer backbone. When linear BuOx was replaced by slightly less hydrophobic but branched *sec*BuOx, a drastic decrease in maximum solubility to 3.6 ± 0.2 g L^−1^ was observed. These results showed unambiguously that the increase of hydrophobicity of micellar core does not lead to increase of loading capacity for this family of polymers and minor structural changes can severely compromise drug loading. The unusually high loading capacity of triblock copolymer pMeOx‐*b*‐pBuOx‐*b*‐pMeOx goes along with interesting changes in the morphology of these micelles. Schulz et al.^[^ ^151^ ^]^ described the morphological changes of ABA triblock copolymers pMeOx‐*b*‐pBuOx‐*b*‐pMeOx and pMeOx‐*b*‐pNonOx‐*b*‐pMeOx in solution caused by the addition of PTX. Without the drug, worm‐like micelles along with spherical micelles were present in both cases, but only spherical micelles remained upon the addition of the drug. These morphological changes were investigated in more details by SANS by Jaksch et al.^[^ ^152^ ^]^ The authors suggested the formation of raspberry‐like particles for pMeOx‐*b*‐pBuOx‐*b*‐pMeOx at intermediate drug loading (33 wt%), but not in case of pMeOx‐*b*‐pNonOx‐*b*‐pMeOx, where the spherical micelles are present. Unfortunately, a detailed analysis of PTX loaded micelles at the highest drug loadings is still pending.

POx are particularly interesting for studying hydrophilic/lipophilic balance for two reasons. First, a large variety of different side chains are easily introduced. Second, and arguably more important, the highly polar tertiary amide in every repeat unit creates a situation where strongly polar hydrogen bond acceptors are present even if longer aliphatic side chains render the polymer water‐insoluble. Accordingly, the hydrophobic core of the micelles allows hydrophobic and polar interactions (H‐bonding) at the same time. POzi give the opportunity for an even more diverse variation of the hydrophilic/lipophilic balance. Accordingly, Lübtow et al. studied formulation of two hydrophobic drugs, PTX and curcumin (CUR) using four different of amphiphilic block copoly(2‐oxazoline)s and copoly(2‐oxazoline)s containing three and four carbons in the side chain, respectively (Figure 10B).^[^ ^153^ ^]^ As previously established, pBuOx‐based formulations were able to solubilize high amount of PTX (drug loading capacity ≈ 50 wt%, solubilization 9 g L^−1^) but failed to solubilize large amounts of CUR. In contrast, its structural isomer comprising pPrOzi as the hydrophobic block did not solubilize PTX well but was an excellent solubilizer for CUR (drug loading capacity > 50 wt%, solubilization 12 g L^−1^). This finding indicates that for specific drug, an optimal chemical composition of the solubilizing copolymer needs to be specified based on mutual interactions. Moreover, a synergistic effect was observed for pPrOzi‐based formulation, when the addition of CUR increased the PTX encapsulation efficiency. On the other hand, the co‐encapsulation of CUR even decreases the loading of PTX in case of pBuOx polymers, indicating an antagonistic effect. In a follow‐up study, the interactions between CUR host molecules and two block copolymers comprising pBuOx (low loading) and pPrOzi (high loading) were studied in more detail by fluorescence spectroscopy.^[^ ^154^ ^]^ Steady‐state fluorescence, fluorescence upconversion, and anisotropy measurements showed a lower molecular mobility of CUR, suggesting stronger interactions between CUR and the hydrophobic micellar core in case of pBuOx formulations, which actually exhibits lower drug loading. This study provides another evidence against the intuitive model of highly hydrophobic micellar core, strongly interacting with hydrophobic drug, being the ideal system to achieve highest loading capacity. A more detailed investigation by the same group^[^ ^21^ ^]^ revealed even higher CUR solubilizations (loading capacity, LC > 50 wt%, solubilization 50 g L^−1^) in case of pPrOzi formulation, by increasing the overall aqueous concentration of similar triblock copolymer in comparison to previous work. Interestingly, this study also revealed that the examined block copolymer did not form micelles without the presence of the drug, due to relatively high solubility of pPrOzi. This finding confirms that the study of self‐assembly behavior of block copolymers without the drugs reveals only partial information and cannot be easily generalized, until the influence of drug molecule is properly considered and tested.

The previously discussed studies focused mostly on the comparison of various lengths of linear aliphatic side chain of POx, or switching of methylene group between side‐chain (POx) and polymer mainchain (POzi). However, POx copolymers represent a rich group with various possible side chain substituents. In this context, Lübtow et al. studied long (C9) branched and linear alkyl chains of both studied types of polymers, POx and POzi, and the effect of chemical composition on loading of the same two model drugs, PTX and CUR.^[^ ^96^ ^]^ The authors hypothesized on the possible impact of crystallization of the side chain on the loading capacity of the micelles, considering that pNonOx is a semicrystalline polymer while side chain branching should impede efficient side chain packing. The authors showed that micelles with the branched poly(2‐(3‐ethylheptyl)‐2‐oxazoline) (pEtHepOx) core‐forming block indeed exhibited higher loading capacities for PTX compared to its linear analogue, pNonOx (5.69 ± 0.72 vs 1.24 ± 0.76 g L^−1^). However, there was an unusual kinetic effect observed. These higher solubilized values in case pEtHepOx were achieved only after 10 days with the apparent solubility increasing over time. Unfortunately, this unusual observation was not studied further and warrants further investigation. In case of the triblocks with either pEtHepOzi or pNonOzi, the difference was less clear and the branched side chains did not give a clear advantage. This may be due to the larger spacing between the side chains. These results seem to be in contrast to an earlier report where branched side chains performed worse, however, in this earlier study, the side chains were much shorter and the branching closer to the amide motif. Also, the loading was still lower than in case of micelles with less hydrophobic BuOx core, which indicates that the side‐chain packing is not the main reason for decreased LC. Side chain crystallization can be ruled out in any case, as the hydrophobic blocks are rather small (≈10 repeat units) and thermal analysis did not reveal any crystallization. Recently, a number of reports suggest the importance of *π*–*π* stacking between drug and drug delivery vehicle to improve stability.^[^ ^155^, ^156^, ^157^ ^]^ Milonaki et al.^[^ ^100^ ^]^ studied the encapsulation of anti‐inflammatory drug indomethacin into gradient copolymers composed of MeOx and aromatic PhOx. The authors achieved quite high loading capacity around 40 wt% and solubilization 3.75 g L^−1^ (recalculated from available data). The authors observed multimodal distribution on DLS with the peaks around 10 and 100 nm (*R*_h_ values), which was explained as co‐existence of micelles and larger aggregates. A possible formation of non‐spherical aggregates was not discussed. Hahn et al. studied in more details the effect of aromatic moieties in hydrophobic POx block on encapsulation efficiency of PTX, CUR, and schizandrin A (SchA, Figure 10C).^[^ ^158^ ^]^ These compounds were selected due to their varying relative aromatic content. While curcumin is relatively small and features a highly conjugated *π*‐electron system with two aromatic rings, PTX is a much larger, predominantly aliphatic molecule with only isolated benzene rings. SchA can be seen somehow in the middle in this respect. The authors showed that the effect of aromatic comonomer (PhOx and 2‐benzyl‐2‐oxazoline, BzOx) core on loading capacity strongly depends on encapsulated drug. In case of PTX, a drug with lowest relative content of carbons in aromatic system, the increase of aromatic comonomer (BzOx and PhOx) led to the decrease of LC. In case of SchA, the compound with intermediate aromatic content, the increase of aromatic comonomer did not have any prominent effect on LC. Finally, for CUR, the presence of aromatic comonomer led to increase of drug loading. In addition, the authors observed somewhat higher LC for BzOx containing copolymers, in comparison to PhOx copolymers, presumably due to the restrained flexibility of the aromatic ring in close proximity of the copolymer backbone. It could be argued that some flexibility should be important in this context, as *π*–*π*‐interactions are rather directionally specific with the displaced and edge‐to‐face orientations being energetically clearly favored over the sandwich orientation.^[^ ^159^ ^]^ Kronek et al. prepared formulations loaded with CUR from gradient copolymer comprising hydrophobic 2‐(4‐dodecyloxyphenyl)‐2‐oxazoline by dialysis method from three different solvents, ethanol, DMSO, and acetonitrile. The maximum achieved drug loading capacity was moderate (22 ± 2%), however, the overall polymer concentration was quite low (4 g L^−1^), leading to quite low solubilizations of CUR (0.08 g L^−1^, recalculated from available data).^[^ ^103^ ^]^ Sedlacek also tested CUR solubilization using gradient copolymers p(MeOzi‐*co*‐BuOx)_*grad*_ but also in this case drug solubilization was poor with 9 wt% CUR solubilized by 2 g L^−1^ gradient copolymer.^[^ ^114^ ^]^ An unusual POx with heterocyclic side chain has been introduced by Hwang et al.^[^ ^94^ ^]^ The diblock copolymer pMeOx‐b‐pcBOx was used to encapsulate several poorly water‐soluble drugs, however, only with mediocre success. The highest loading capacity, 25 wt% (solubilization 3.28 g L^−1^), was achieved for LY2109761, a transforming growth factor *β* (TGF‐*β*) receptor inhibitor. Salgarella, Zahoranová et al. studied encapsulation of anti‐inflammatory drug dexamethasone (DEXA) into five different diblock and triblock copoly(2‐oxazoline)s.^[^ ^160^ ^]^ Depending on the polymer structure, they observed formation of small micelles (diameter 35 nm), but also larger aggregates (hundreds of nm—several microns). The loading capacity ranged between 4.2 wt% (solubilization 0.44 g L^−1^) in case of copolymer p(EnOx‐BuOx)_stat_‐*b*‐pMeOx to 14.7 wt% (solubilization 1.72 g L^−1^) in case of diblock copolymer pPrOx‐*b*‐pMeOx. Even though the highest achieved loading capacity is relatively low comparing to the POx and POzi formulations comprising CUR or PTX discussed above, it is still higher than DEXA formulations based on other block copolymers.^[^ ^161^, ^162^, ^163^, ^164^ ^]^ Interestingly, also other POx, including pBuOx and pPrOx containing block copolymers with lower DP and different ratio of blocks exhibited lower loading capacities toward DEXA^[^ ^99^ ^]^ These effects of copolymer composition require deeper examination. The release of DEXA from the micelles was accelerated by using ultrasound (US) as an external stimulus, however, the study was performed only in vitro using dialysis cups. There is evidence that the use of US combined with administration of doxorubicin encapsulated in Pluronic micelles decreased the size of tumors significantly more that administration of doxorubicin in micelles without US‐treatment, as tested in vivo in rats.^[^ ^165^ ^]^ It can be hypothesized that similar effects may be possible for POx‐based micelles.

Apart from experimental approaches, several research groups attempted the theoretical prediction of drug–copolymer interactions and hence, solubilization. A block copolymer composed of EtOx and EnOx was studied by Dargaville et al.^[^ ^166^ ^]^ for the formulation of CUR. The authors rationalized the selection of the hydrophobic block based on Hansen solubility parameters (HSP) and Flory–Huggins parameters, calculated a group contribution method (van Krevelen method), which provides values of dispersion, polar, and hydrogen bonding component for each structural unit in the molecule. However, the achieved loading capacity was rather low with 12 ± 2 wt% (solubilization 0.04 g L^−1^, as recalculated from available data), and maximum solubility much lower (about 1000‐fold) compared to other POx/POzi‐based triblock copolymer formulations with solubilization of more than 50 g L^−1^ and >50 wt%.^[^ ^21^ ^]^ Lübtow et al. also attempted to correlate HSP (and corresponding Flory–Huggins interaction parameters) of a small library of 18 different POx and POzi triblock copolymers with 5 different drugs.^[^ ^99^ ^]^ In addition to theoretically calculated parameters, the authors introduced experimental determination of HSP from experimental solubility for the drugs and block copolymers in a selection of 31 different solvents. The authors showed that HSP correlated better with the drug encapsulation than Flory–Huggins parameter, but overall, the correlation was not entirely satisfying. Nevertheless, large *R*_a_ values (corresponding to a large difference in HSP values) did correlate reasonably well with poor solubilization, and the best solubilizers typically also yielded minimal *R*_a_ values. However, due to the conceptual limitations of theoretical HSPs,^[^ ^167^ ^]^ these cannot differentiate well between very similar polymers, especially structural isomers. Likewise, the approach to use experimentally determined HSP confounded the influence of the solubility the different blocks in the block copolymers. It would be interesting to see the correlation of the drug solubilization and the HSP of the hydrophobic blocks alone, but again, this is not likely to describe the complex situation in micellar drug formulations well, as will be discussed below in more detail. Recently, Tropsha, Kabanov et al.^[^ ^168^ ^]^ have developed a computational quantitative structure‐properties relationship model to predict loading capacities of drug‐POx formulations. The model was based on experimental data of encapsulation of 41 different drugs into triblock and diblock POx containing BuOx, NonOx, and BzOx, with MeOx as hydrophilic block, taking into account the chemical structure of copolymers and drugs, as well as formulation conditions. While a great number of different drugs were considered within the model, a larger structural diversity of copolymers will be interesting to consider in follow‐up work. Using the developed model, the authors were able to accurately predict the loading capacities for six out of eight newly tested drugs.

#### 3.1.2 Interactions of Drug with Micellar Corona

It is generally considered that the encapsulated hydrophobic drug is located mainly or exclusively in the hydrophobic core of the polymeric micelles. However, detailed investigations by Stenzel and co‐workers using glycopolymer micelles loaded with platinum complexes, CUR, or PTX revealed the presence of loaded drug also within hydrophilic corona, affecting its structure and hydration state.^[^ ^169^, ^170^ ^]^ Furthermore, the cellular uptake was also affected by the level of drug loading within the micelles, with higher loading capacities leading to lower micelles uptake by cancer cells.^[^ ^171^ ^]^

**Figure 11:**
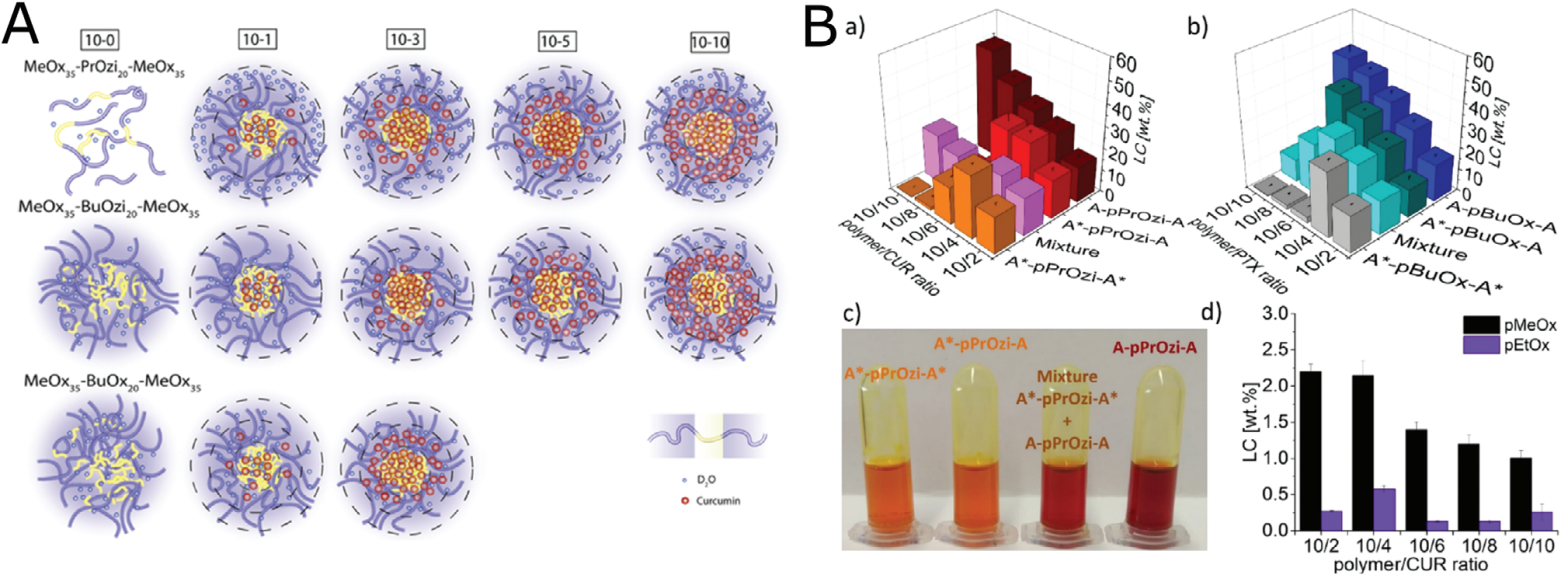
Interactions of drug with micellar corona. A) Schematic illustration of the different micellar morphologies at various CUR contents shown. The sizes of the micelle compartments are not to scale to facilitate comparability. To visualize the amount of CUR in each micellar section, the number of red dots roughly represents the respective CUR concentration. Reproduced with permission.^[^ ^146^ ^]^ Copyright 2020, American Chemical Society. B) Achieved loading capacity at different ratios (w/w in g L^−1^) of a) CUR and b) PTX and triblock co‐ and terpolymers with pPrOzi and pBuOx as the hydrophobic block and four different setups of hydrophilic blocks, that is, A−B−A, A*−B−A, A*−B−A*, and a mixture (A−B−A and A*−B−A* 1:1 w/w) (A = pMeOx and A* = pEtOx). c) Visual appearance of CUR aqueous formulation prepared with four different setups of hydrophilic blocks at the polymer/CUR feed ratio of 10/2 g L^−1^. d) CUR‐solubilizing capacity of the corona‐forming blocks as homopolymers pMeOx (black) and pEtOx (violet). In all cases, the polymer feed was 10 g L^−1^, and the drug feed was 0 to 10 g L^−1^. Data is given as means ± SD (*n* = 3). Reproduced with permission.^[^ ^174^ ^]^ Copyright 2020, American Chemical Society.

Also, in case of POx and POzi micelles, the interactions of drugs with micellar corona were recently investigated. The internal structure of CUR‐loaded micelles prepared from triblock ABA copolymers (A = pMeOx, B = pBuOx, pPrOzi, or pBuOzi) were studied by Sochor et al. using SANS.^[^ ^146^ ^]^ The authors observed differences between high‐loading and low‐loading capacity formulations (**Figure** **11**A) and proposed a core–shell–shell model of the micelles with high‐loading capacities, corresponding to drug incorporated into micellar corona forming essentially a second shell. Further, Pöppler et al.^[^ ^172^ ^]^ investigated the role of hydrophilic block in more details using solid‐state NMR, using the triblock copolymer pMeOx‐*b*‐pPrOzi‐*b*‐pMeOx, again loaded with CUR as a model compound. The authors proposed a structural model according to which the corona‐forming hydrophilic blocks start to interact with the drug at higher drug loading. This supposedly allows the unusually high loading capacities, which cannot be reasonably explained by loading of the hydrophobic core alone. However, as the hydrophilic polymer interacts with the drug at higher drug loading, its ability to undergo H‐bonding with water molecules is impeded, which resulted in lower dissolution rates of freeze‐dried micellar formulations. Once dissolved, the drug loaded polymer micelles showed excellent stability, but as the dissolution rates were quite strongly affected, an application as dried powders for oral administration would probably require additional dissolution aides or lower drug loading. More recently, solid‐state NMR spectroscopy was also used to study in more detail the internal structure of pMeOx‐*b*‐pBuOx‐*b*‐pMeOx loaded with PTX. Specifically, ^14^N‐^1^H heteronuclear multiple‐quantum correlation (HMQC) solid‐state NMR was employed to study the interactions of amide groups in polymer and drug molecules.^[^ ^173^ ^]^ In comparison to neat polymer and PTX, the formulations exhibited decreasing in ^14^N quadrupolar shift at negative values, which suggests a more symmetric nitrogen environment. The authors assigned these spectral changes to the tertiary amides of POx in a close proximity of PTX molecules serving as hydrogen bond acceptors, leading to the more symmetric nitrogen environment. Whether this occurs only in the micellar core or the micellar corona was not discussed in this contribution. However, this work highlights the potential of ^14^N‐^1^H HMQC NMR to unravel the interactions between polymers and loaded compounds.

Finally, the impact of hydrophilic corona of ABA‐type triblock copolymers on the encapsulation of CUR and PTX was also studied by variation of the structure of the hydrophilic corona by Haider et al.^[^ ^174^ ^]^ The authors compared two different hydrophilic blocks, the previously heavily investigated pMeOx and the more amphiphilic pEtOx. pEtOx comprising formulations exhibited significantly lower loading capacities compared to pMeOx‐based formulation.

It appears that this can be attributed to a stronger interaction between pEtOx corona and drugs, leading to micellar aggregation and therefore, colloidal instability (Figure 11B). Interestingly, this mirrors the previous report from the same group, where it was found that a stronger interaction of the drug with the micellar core was also found to be detrimental to drug loading.^[^ ^154^ ^]^

### 3.2 In Vitro and Vivo Safety and Efficacy of Drug Formulations

As it was discussed in previous sections, POx‐ and POzi‐based block copolymers proved to be efficient solubilizers for various hydrophobic drugs, with drug loadings routinely well above the limit of 25–30 wt% commonly observed for polymer micelles. As discussed, this seems to be connected with the involvement of the highly polar MeOx repeat units in the interaction with the drugs. However, the unusual high drug loading does bring another important question; whether these high loading capacities are translated into improvement of drug efficacy and safety in vitro and in vivo. This will be addressed in following paragraphs.

POx‐based polymers have been considered an alternative to PEG for several decades, in particular the hydrophilic pMeOx and pEtOx. Accordingly, it has been established early that generally speaking, pMeOx and pEtOx are very cytocompatible,^[^ ^20^, ^23^ ^]^ show low unspecific organ uptake,^[^ ^31^, ^175^ ^]^ low uptake in the organs of the so‐called reticulo‐endothelial system,^[^ ^176^ ^]^ and are readily cleared via the kidneys if small enough.^[^ ^25^ ^]^ However, amphiphilic block copolymers must be considered with additional scrutiny, as their physico‐chemical characteristics can resemble lipopolysaccharides, which in turn can trigger an immune response via the complement system.

**Figure 12:**
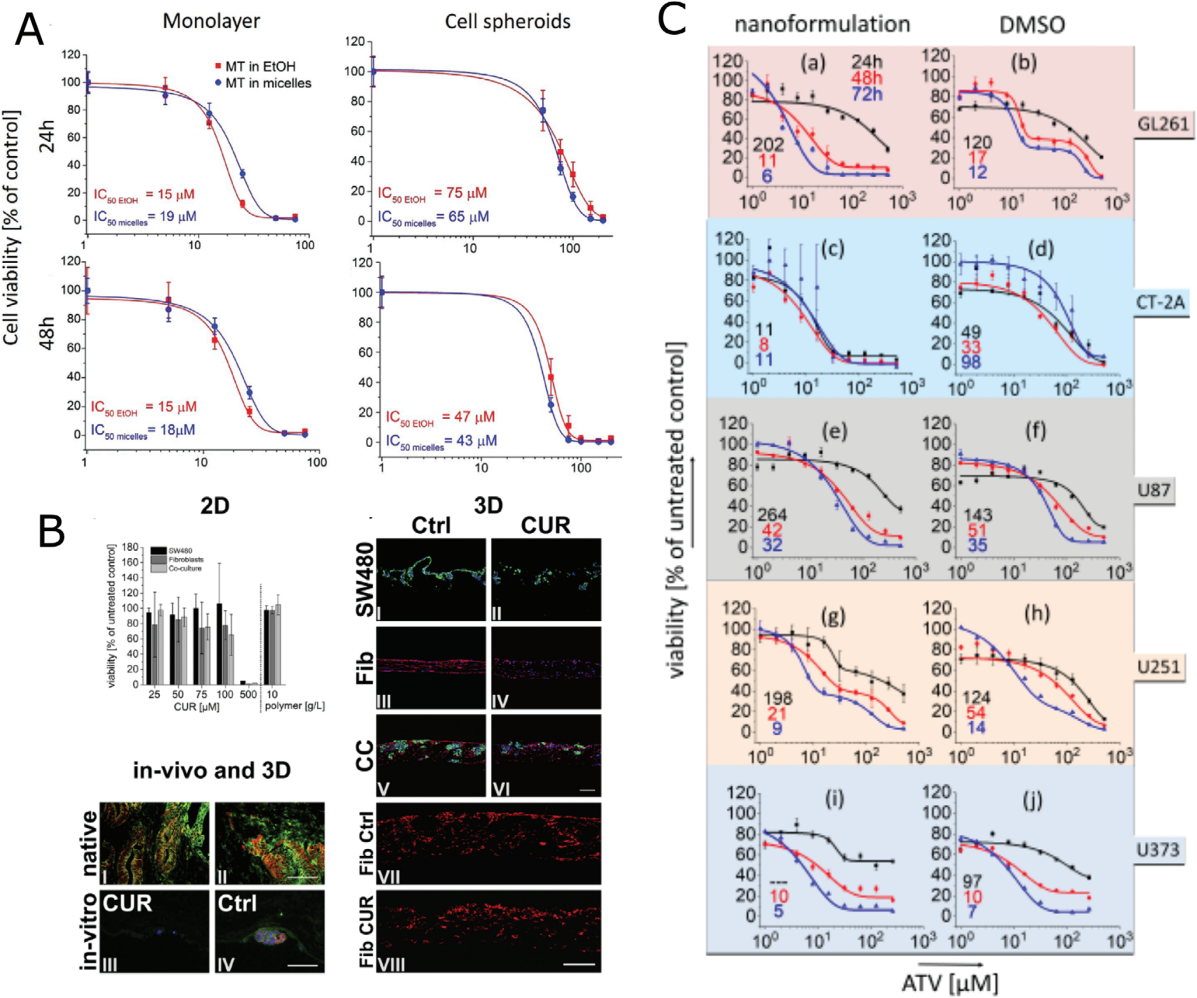
In vitro tests using POx‐based formulations. A) Cell viability and corresponding IC_50_ values of mitotane dissolved in ethanol (red) and as micelles formulation (blue) in NCI‐H295R monolayer after incubation for 24 and 48 h and 3D tumor spheroids 24 and 48 h as determined by CellTiterGlo assay. Monolayer and tumor spheroids were treated with the range of mitotane concentrations dissolved in ethanol and as A‐pBuOx‐A nanoformulation. All the values are average of replicates expressed relative to cell viability values in control cells normalized to 100%. Data points represent average of *n* = 3 experiments with eight technical replicates per mitotane concentration for each experiment ± standard deviation. Reproduced with permission.^[^ ^177^ ^]^ Copyright 2019, Wiley‐VCH GmbH. B) A‐pPrOzi‐A‐formulated CUR (50 µm) leads to reduced SW480 cell numbers under 3D but not 2D conditions and has anti‐metastatic effects in a metastasis model of colorectal cancer. In 2D, CUR has no significant effects on the viability of cells in mono‐culture of SW480 cells up to 100 µm, while the viability of mono‐cultured fibroblasts as well as the co‐cultured fibroblasts with SW480 was somewhat diminished. Pan‐Cytokeratin (green)/Vimentin (red) stainings of static 3D models. CUR diminishes tumor cell numbers in mono‐ and co‐cultures as shown in I and II as well as V and VI. Fibroblasts in mono‐culture are affected by CUR and changed their morphology as shown in III and IV. In contrast, cocultured fibroblasts did not change their morphology after treatment as shown in VII (untreated control) and VIII (treatment with CUR). Fib = fibroblasts, CC = Co‐culture, Ctrl = untreated control, CUR = A‐pPrOzi‐A‐formulated CUR. Scale bar in VI = 100 µm for I to VI, scale bar in VIII = 100 µm for VII and VIII. c) I: In a native non‐invasive tumor, E‐Cadherin (green) and *β*‐Catenin (red) are co‐localized at the cell boundaries. II: In an invasive carcinoma, *β*‐Catenin translocates to the cytoplasm and into the nucleus. III: SW480 cells in the flowing medium of a flow bioreactor are hampered to adhere after CUR treatment and express neither *β*‐Catenin nor E‐Cadherin. IV: Untreated SW480 cells adhere to the matrix SISmuc in a flow bioreactor displaying *β*‐ Catenin in the cytoplasm and the nucleus as well as E‐Cadherin at the cell boundaries of the tightly packed cells. Scale bar in II = 100 µm for I and II. Scale bar in IV = 100 µm for III and IV. Reproduced with permission.^[^ ^21^ ^]^ Copyright 2019, Elsevier B.V. C) Concentration‐dependent cell viability of A‐pBuOzi‐A/ ATV (left column) or DMSO/ATV (right column) against mouse glioma cells a,b) GL261 and c,d) CT‐2A, or e,f) human glioblastoma cells U87, g,h) U251, and i,j) U373. Cell viability was determined (CCK‐8 assay) after 24 h (black), 48 h (red), and 72 h (blue) ATV treatments. IC_50_ values for 24 h (black), 48 h (red), and 72 h (blue) are given in µm ATV in the bottom left corner of each graph. Data are presented as mean ± SD (*n* = 3 (individual 96‐well plates) × 4 (wells per 96‐well plate)). Vehicles were used as a control. Reproduced with permission.^[^ ^178^ ^]^ Copyright 2020, American Chemical Society.

Haider et al. developed mitotane formulation for potentially improved treatment of adrenocortical carcinoma.^[^ ^177^ ^]^ The authors screened a small library of different POx and POzi triblock copolymers and selected pMeOx‐*b*‐pBuOx‐*b*‐pMeOx based on its highest loading capacity 36 wt% (solubilization 5.71 ± 0.11 g L^−1^). However, the loading capacity dramatically decreased after 24 h of storage, caused by drug precipitation. The cytotoxicity of formulations compared to mitotane in ethanol was tested on NCI‐H295R human adrenocortical carcinoma cell lines. In 2D cell monolayer, no difference in toxicity of mitotane formulated in micelles compared to ethanolic drug solution was observed (50% inhibitory concentration IC_50,24h_ = 15 and 19 µM for mitotane dissolved in ethanol and micelles, respectively). As an improved in vitro model, potentially somewhat more appropriately reflecting in vivo conditions, 3D spheroids from the same cell line were used. As often observed, the spheroids exhibited higher tolerance toward drug, with IC_50,24h_ values increasing to 75–65 µM for mitotane in ethanol and in micelles, respectively (**Figure** **12**A). Although these values cannot be considered statistically different either, it is interesting to consider that the increase of IC_50_ values between the 2D and 3D model was fivefold for the ethanolic mitotane solution, while the IC_50_ value increased only by a factor of 3.4 for the micellar formulation. Mitotane is clinically used in form of Lysodren tablets. This formulation suffers from severe shortcomings in terms of bioavailability and pharmacokinetics. The promise of this novel nanoformulations would be that either bioavailability could be increased significantly, or mitotane could even be administered intravenously, which potentially could provide major benefits to patients. However, this remains to be established.

Atorvastatin is another drug that is widely used in clinical practice. Lübtow et al. developed nanoformulation of atorvastatin for potential treatment of glioblastoma.^[^ ^178^ ^]^ For the treatment of dyslipidemia, Atorvastatin is taken orally, however, the serum concentrations necessary for glioblastoma treatment are unlikely to be achieved with the commonly used dosage form and this route of administration. The authors tested several ABA triblock copolymers with MeOx as hydrophilic block (A), varying in hydrophobic block (B = PrOx, BuOx, PrOzi, and BuOzi). The copolymer pMeOx‐*b*‐pBuOzi‐*b*‐pMeOx was selected based on highest exhibited loading capacity 41 wt% (solubilization 6.9 g L^−1^). In vitro cytotoxicity of the prepared micellar formulation against various glioblastoma cell lines in 2D, as well as in spheroids, was tested (Figure 12C). While in different 2D monolayer cell cultures, no conclusive differences between micellar formulation and drug dissolved in DMSO could be seen, in spheroid models, micellar formulations were more active compared to atorvastatin in DMSO. It is worth noting that the micellar formulation with atorvastatin also proved to be more effective compared to free drug solution against glioblastoma stem cells, which could have implications for cancer relaps. In addition to cytotoxicity tests, in vitro permeation of the micellar formulation through a blood‐brain‐barrier (BBB) model based on human induced‐pluripotent stem cells. However, the membrane permeation of the drug was not improved using micellar formulation. Nevertheless, the nanoformulations could enable plasma concentrations higher than the current dosage form and potentially sufficient for glioblastoma treatment or adjuvant therapy, but this will have to be established in a suitable in vivo model.

As mentioned previously, the triblock copoloymer pMeOx‐*b*‐pPrOzi‐*b*‐pMeOx has a particularly high loading capacity for CUR.^[^ ^21^ ^]^ The effect of these CUR nanoformulation on 2D and 3D cultures of breast and colorectal cancer was studied. In accordance with previously discussed results, no significant differences in cytotoxicity between micellar formulation and CUR in DMSO was observed in 2D cancer cell models. To mimic better the in vivo environment, the authors employed a 3D cancer model, derived from decellularized porcine jejunum (small intestinal submucosa with preserved mucosa, SISmuc matrix) seeded with breast cancer cells. Again, the 3D breast cancer model exhibited higher resistance toward CUR compared to 2D models, since the dose of CUR needed to induce cytotoxic effect on breast cancer cells was 100 µm, in comparison to 15 µm in case of 2D cancer cells. In case of 3D model of colorectal cancer, the authors used the same matrix, but co‐cultivated colorectal cancer cells with fibroblasts, to induce invasive tumor cell growth. Interestingly, the results were opposite for the more complex colorectal cancer model in comparison to breast cancer model; here the cancer cells were more sensitive to CUR in 3D co‐culture (Figure 12B). It should be noted that here, also in case of 3D models, not much difference was observed between CUR solubilized in micelles and DMSO, respectively, except for a better solubility at high concentrations in case of micellar formulation. From DMSO solution, the CUR precipitated at higher concentrations, while no precipitation was observed for the nanoformulations. This is not a trivial difference, as precipitation in vivo could of course lead to severe issues.

Ubiquitin carboxyl‐terminal hydrolase L1 (UCHL1) is a multifunctional protein and enzyme, which is believed to play a role in cancer metastasis. For this reason, small molecular inhibitors of de‐ubiquitinating activity of UCHL1 are in center of interest for the development of potential therapies. Shakelford et al. prepared formulation of LDN‐57444, one of such promising inhibitors, encapsulated into the same pMeOx‐*b*‐pBuOx‐*b*‐pMeOx triblock for the treatment of oral and nasopharyngeal carcinoma.^[^ ^179^ ^]^ Interestingly, in contrast to other works previously discussed here, the ultimate goal was not to induce cytotoxic effects, but to inhibit UCHL1. For this reason, lower concentrations (3 µm) of the formulated inhibitor were used. The effect of micellar formulation and free LDN were studied in vitro and the authors could show that both forms of LDN reduced migration of UCHL1‐possitive cells, eventually decreasing the metastatic potential. Interestingly, the micellar formulations appeared to be more effective than free inhibitor against nasopharyngeal NP69 cell line, but such differences were not observed in the case of oral squamous carcinoma cells. The explanation of this selective effect would require further study.

In recent years, major advances in in vivo evaluation of POx‐based micelles have come out of the Kabanov lab, although mostly using the triblock copolymer pMeOx‐*b*‐pBuOx‐*b*‐pMeOx, which was the first POx copolymer found to exhibit ultra‐high drug loading capacities. A formulation of PTX solubilized with triblock copolymer pMeOx‐*b*‐pBuOx‐*b*‐pMeOx exhibiting high loading capacity was characterized and tested in vivo for safety assessment and treatment against breast and ovarian cancer.^[^ ^180^ ^]^ As mentioned before, the PTX/POx formulation exhibits significantly higher loading capacity and maximal PTX concentration compared to clinically approved formulations, Taxol (PTX formulated with excipient Cremophor EL/ethanol) and Abraxane (PTX formulated with human serum albumin). Prior to in vivo experiments, the authors studied the interactions of PTX/POx formulation with serum proteins. At higher PTX concentrations (2 g L^−1^), more than 80% of drug was still encapsulated in micelles, even at the presence of serum, in comparison of only 20% in the case of Taxol.

Next, in vivo toxicity of the formulation was evaluated on mice. It was shown that the maximum tolerated dose (MTD) of PTX/POx formulation was 150 mg kg^−1^, in comparison to much lower MTD in case of Taxol (20 mg kg^−1^) and Abraxane (90 mg kg^−1^). Finally and most importantly, antitumor efficacy was tested in various tumor models, including orthotopic, syngeneic models which reflect the poor response to chemotherapy observed in the clinical situation of triple‐negative breast cancer to evaluate the therapeutic effect of the formulations. The POx micellar formulations outperformed both formulations Taxol and Abraxane leading to improved tumor growth inhibition and prolonged survival of mice.

**Figure 13:**
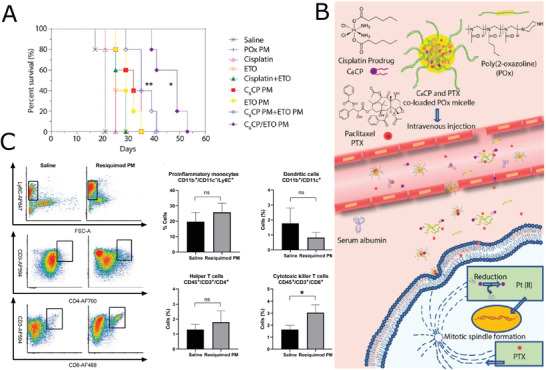
In vivo investigations using POx formulations. A) Kaplan−Meier survival plot showing antitumor effects of the single and co‐loaded drug PM in 344SQ/Luc. NCSLC animal model. The treatments regimen was q4d × 4. Drug injection doses were: 30 mg kg^−1^ of ETO and 15 mg kg^−1^ C6CP for C6CP/ETO PM (4/8/10) and mixture of C6CP PM (4/10) and ETO PM (8/10); 30 mg kg^−1^ ETO for ETO PM (8/10), 15 mg kg^−1^ C6CPs for C6CP PM (4/10); 2 mg kg^−1^ cisplatin or 4 mg kg^−1^ ETO for free drugs; 2 mg kg^−1^ cisplatin and 4 mg kg^−1^ ETO for free drugs mixture. Empty PM were injected at the polymer dose equivalent to that in the co‐loaded micelle formulation. *p < 0.05 (vs C6CP PM and ETO PM mixture group), **p < 0.01 (vs C6CP PM group). Reproduced with permission.^[^ ^181^ ^]^ Copyright 2018, American Chemical Society. B) The strategy using POx micelles for the co‐delivery of PTX and CP prodrug. Reproduced with permission.^[^ ^182^ ^]^ Copyright 2018, Elsevier Ltd. C) Resiquimod PM induces TH1 polarization of immune cells in the TME. Representative fluorescence‐activated cell sorting (FACS) plots of CD11b+/ CD11c−/Ly6C+, CD45+/CD3+/CD4+, and CD45+/CD3+/CD8+ cell populations from the tumors of mice treated with saline and Resiquimod PM. FSC‐A: forward scatter area. Quantification of the indicated population of cells. Data represent means ± SEM. *n* = 4. *p < 0.05 computed by unpaired Student's *t*‐test with Welch's correction. Significance level *α* was set at 0.05. ns, not significant. Reproduced with permission.^[^ ^185^ ^]^ Copyright 2020, American Association for the Advancement of Science (AAAS).

More recently, Kabanov et al. tested the same formulation based on pMeOx‐*b*‐pBuOx‐*b*‐pMeOx triblock copolymer for the co‐delivery of etoposide (ETO) and cisplatin (C_6_CP) for the treatment of lung cancer.^[^ ^181^ ^]^ The obtained loading capacities of the single drug formulations was 50 wt% (solubilization 9.7 g L^−1^) for ETO and loading capacities 45−46% (solubilization 8.4 g L^−1^) for cisplatin complexes. In case of co‐formulations, the total solubilization of both drugs was 11 g L^−1^ what is higher than solubilization of each drug separately. Interestingly, the co‐loaded micelles exhibited worm‐like morphology, while the empty micelles exhibited spherical shape. In vivo tests were performed in nude mice bearing A549 and H69AR (human lung carcinoma cell lines) xenograft and 344SQ orthotopic lung cancer model. To evaluate the therapeutic efficacy of the formulations, the tumor volume development and animal survival rate was monitored. The authors observed slower increase of tumor volume in case of micellar formulation of C_6_CP and ETO, in comparison to free drug formulation. Even more surprisingly, micelles co‐loaded with C_6_CP and ETO outperformed co‐administered mixture of single drug‐loaded micelles. Similar trends were observed for the prolonged survival of mice (**Figure** **13**A). In a follow‐up study, the authors employed similar strategy for co‐delivery of different combination of drugs, PTX and cisplatin encapsulated in the same triblock pMeOx‐*b*‐pBuOx‐*b*‐pMeOx for the treatment of ovarian and breast cancer (Figure 13B).^[^ ^182^ ^]^ The studies on ovarian cancer cells in vitro reveals a synergistic effect of co‐loaded micelles. In vivo studies revealed, in accordance with previous study, significantly suppressed tumor growth ration and increased survival of mice, in comparison to free drug formulation, but also in comparison to a mixture of single‐drug loaded micelles. This improved therapeutic outcome of micelles with drug combinations versus combinations of micelles loaded with a single drug each is very notable. This shows that the altered structure and dynamic from the co‐loading (drug/drug and drug/polymer interactions) in co‐loaded micelles in fact translate into a noticeable beneficial effect in the complex in vivo situation.

Further, the encapsulation of vismodegib in POx‐based micelles for the treatment of medulloblastoma was studied in a mouse model.^[^ ^183^ ^]^ Formulation into POx micelles led to increased MTD and better penetration into brain and tumor compared to conventional vismodegib formulation. Notably, this stands in contrast, as previously discussed, to in vitro results where POx micelles were not shown to improve the permeation of drug through an in vitro blood‐brain‐barrier model, albeit for a different drug (atorvastatin).^[^ ^178^ ^]^

Stimulating of the own immune system of a patient is a promising strategy in cancer treatment.^[^ ^184^ ^]^ An encapsulation of Resiquimod, an immune response modifier, into pMeOx‐*b*‐pBuOx‐*b*‐pMeOx micelles has been investigated for immunotherapy of non‐small cell lung cancer.^[^ ^185^ ^]^ As a comparison, conventional anticancer drugs, such as cisplatin and PTX, were also formulated with POx. Interestingly, the micellar formulations of the conventional drugs exhibited in most cases lower cytotoxicity in vitro in comparison to free drugs, and in this case, they did not lead to improved survival in vivo, in comparison to control group treated with saline. On the other hand, POx/Resiquimod formulation led to significant increase of survival in the rodent tumor model. This anticancer effect was supposedly caused by stimulation of anticancer immune response, rather than a direct cytotoxic effect towards cancer cells (Figure 13C).

In summary, the unparalleled high drug loading capacity of POx/drug formulation are a strong motif for the exploitation of these carriers in vivo. The therapeutic outcomes of single drug or drug combination formulations in rodent tumor models are very promising and seem to be enabled, at least in part, by the high drug loading and the possibility to effectively co‐load different drugs. In addition to formulation with cytostatic drugs, also another types of therapeutics, such as immune‐response modifiers and inhibitors of metastatic activity seem to benefit from POx micellar carriers. Important to note, while the POx formulations perform excellently in vivo, in vitro, particularly in traditional 2D cell cultures, the potential benefits of these nanoformulations can typically not be fully appreciated.

## 4 Conclusions and Outlook

In conclusion, POx and POzi are two closely related polymer families with a tremendous potential in a variety of applications. They offer a huge molecular toolbox, which we have only started to utilize more comprehensively. Compared to many other polymers, the tertiary amide at the polymer backbone is a particularly interesting feature offering robust polar interactions even in an otherwise hydrophobic environment. While for many decades only few members of these two families have been studied in detail, the last few years have seen a very significant increase in reports on POx/POzi‐based self‐assemblies. Much work has been done in the field of thermoresponsive POx copolymers, where new architectures, such as gradient or star‐shaped copolymers have been studied in a great detail, with respect to control over LCST, temperature driven self‐assembly, crystallization, or gelation. While thermogelling POx were shown to be suitable candidates for bioinks or biomaterial inks for 3D printing, the application potential of other LCST copolymers or thermoresponsive stars or micelles remains more vague despite them being much more established in research. Although such materials are often discussed as potential stimuli‐responsive drug carriers, the effect of encapsulated drug on the thermoresponsive behavior, sensitivity to salt and other co‐solvents makes their application in complex biological media challenging. The inclusion of thermoresponsive POzi may add some additional design space but if this can increase the prospect of real‐life applications remains to be seen. Also, gradient copolymers are gaining more and more attention recently. While some interesting features of this class of copolymers have been described, much more is still waiting to be discovered. It will be interesting to see in which applications gradient copolymers can outperform or at least compete with block copolymers so that one can really benefit from the somewhat simpler preparation. It will be interesting to see more of direct comparisons between of gradient and block POx (POzi) with similar chemical composition with respect to physico‐chemical properties, self‐assembly, and drug loading, but also the comparison of the performance of these two types of copolymers in vitro and, more importantly, in vivo. Also, a wider monomer base for the preparation of gradient copolymers is warranted and expected in the near future. As outlined in this review, one particular promising and heavily investigated application is the use of polymer self‐assemblies for biomedical applications, be it the formulation of hydrophobic drugs, complexation of RNA/DNA, or the use of stimulus‐responsive and cytocompatible hydrogels for tissue engineering and biofabrication. While several formulations of different drugs with different POx and POzi, exhibiting extraordinary high loadings, have been studied in vitro in 2D and 3D cell models, in vivo test were done almost exclusively on one specific pMeOx‐*b*‐pBuOx‐*b*‐pMeOx block copolymer, but this can also be expected to change very soon. It has been shown in vitro, that other block copolymers can have even higher drug loading and therefore, other POx and POzi copolymers may be beneficial for different therapeutic applications. Both scale‐up and production of POx under GMP have been demonstrated, so no obvious fundamental roadblocks are in the way of further development of POx (and POzi) based biomaterials. However, long‐term safety is certainly an issue which will have to be looked at in the future.

With first clinical trials under way, and many more actors starting to work with POx both in academia and industry, it will be very exciting to see the developments in the next few years, which we strongly believe will be highly dynamic.

## Conflict of Interest

R.L. is a co‐founder and interested in the commercial success of DelAqua Pharmaceuticals Inc., which has the intent of developing of polymeric micelle drug formulations. R.L. is co‐inventor on several patents pertinent to the subject matter. The other authors have no competing interests to report.
